# In vitro and in vivo studies of selenium nanoparticles coated bacterial polysaccharide as anti-lung cancer agents

**DOI:** 10.1186/s12934-024-02601-z

**Published:** 2024-12-19

**Authors:** Nourhan S. Shehata, Bassma H. Elwakil, Salma S. Elshewemi, Doaa A. Ghareeb, Zakia A. Olama

**Affiliations:** 1 Department of Medical Laboratory Technology, Faculty of Applied Health Sciences Technology, Pharos University in Alexandria, Alexandria, Egypt; 2 Department of Botany and Microbiology, Faculty of Science, Alexandria University, Alexandria, Egypt; 3 Zoology Department, Faculty of Science, Alexandria University, Alexandria, Egypt; 4 Bio-Screening and Preclinical Trial Lab, Biochemistry Department, Faculty of Science, Alexandria University, Alexandria, 21526 Egypt

**Keywords:** Exopolysaccharide, Antimicrobial, Anticancer, Optimization, Selenium nanoparticles coated bacterial polysaccharide

## Abstract

Microbial exopolysaccharides (EPSs) possess valuable biological functions and fascinating physicochemical properties. On the other hand, lung cancer is the primary contributor to global cancer-related deaths. However, health and safety concerns have prevented the identification and approval of any medications, including chemotherapeutic agents, for lung cancer treatment to date. The current study aims to enhance the production of bacterial EPS as a coating agent for the synthesis of selenium nanoparticles (AZEPS-SeNPs), to enhance their biological activity against pathogenic microbes, human lung adenocarcinoma cells (A549) in vitro, and diethyl nitrosamine (DEN)-induced lung cancer in vivo. The synthesized AZEPS-SeNPs exhibited a significant antifungal effect reaching 49.3 mm against *Candida albicans*. SeNPs and EPSs demonstrated a concentration-dependent synergistic antioxidant effect of 96.8%. Moreover, the synthesized nanoparticles showed a highly potent cytotoxic effect against A549 cells (1.724 ± 0.08 µg/mL) with a therapeutic index of 7.18 ± 0.21 that leads to increased reactive oxygen species (ROS) production. AZEPS-SeNPs demonstrated a proapoptotic effect on the lung adenocarcinoma A549 cell line by stimulating caspase 3 and Bax (7.08-fold and 6.505-fold, respectively), inhibiting the anti-apoptotic gene Bcl2, and arresting the cell cycle in the S phase. In vivo study revealed that the AZEPS-SeNPs-treated group showed improved histopathological examination of lung tissue sections. The present study concluded the efficiency of the synthesized bacterial EPS-SeNPs as multi-functional antimicrobial, anticancer and antioxidant agent.

**Supplementary Information:**

The online version contains supplementary material available at 10.1186/s12934-024-02601-z.

## Introduction

Lung cancer is a prevalent kind of cancer and a major cause of cancer-associated fatalities globally. Regarding its elevated mortality rate and prevalence, most lung cancer cases (85%) are classified as non-small cell lung cancer (NSCLC), which is typically treated with chemotherapy, surgery, radiation, immunotherapy, and targeted therapy.

A benign alternative lung cancer treatment was offered by applying several innovative natural bioactive compounds of microbial origin with more beneficial effects in treating cancer compared to synthetic drugs. Natural polysaccharides have succeeded as a glamorously proposed solution and are considered as generally safe compounds. Moreover, natural polysaccharides are synthesized by a wide range of microorganisms [1]. Microbial polysaccharides were constructed in response to different external factors or to develop protective biofilms, depending mostly on the microbial natural source and nutrients utilized. Exopolysaccharides (EPS) represent multifunctional polymer resources with several applications in the pharmaceutical, flocculant, additive, preservative, petroleum, environmental, and medical fields as anticancer, antioxidant, antiviral, and drug delivery agents [2, 3].

β-(2, 6) fructofuranosyl links are predominately the backbone of microbial levan, which is considered one of the most promising microbial exopolysaccharides. It has gained considerable biomedical interest due to its moisturizing characteristics, good biocompatibility, minimal cell cytotoxicity, antioxidant, antimicrobial, anti-cancer, and anti-inflammatory characteristics [4, 5].

The improvement of polysaccharide production rate was applied using Taguchi’s approach to statistically optimize the produced EPS, this design is considered superior to other statistical methods for enhancing the efficiency and reproducibility of the experimental trials and reducing the possible errors [4]. Currently, nanotechnology is used in medical sciences as nanomedicine, which enables the safe delivery of treatments to reduce their side effects and enhance the drug absorption and bioavailability [6–8].

According to Hertadi et al. [9], it was proved that levan had the potential to be utilized in metal ion nanoparticles (NPS) production that improved the NPS properties as adhesion strengths, larger surface area, and enhanced bioreactivity. It was reported that Levan has a unique ability to undergo self-assembly and transform into nanoparticles when it encounters water, which makes it a perfect candidate for drug delivery in the biomedicine field [10].

On the other hand, for biological processes, selenium (Se) is a vital trace mineral, responsible for selenoenzyme synthesis, which regulates the human body’s physiological functions [1]. Se is also a significant factor in immune, anticancer, catalytic, anti-inflammatory, and antioxidant processes. Conversely, high Se consumption may lead to negative consequences [11].

Research has been conducted on selenium nanoparticles (SeNPs) green production to minimize the possible toxicological issues associated with Se high intakes and improve its bioavailability and biological activity significantly. It was revealed that EPS can be employed as a stabilizing and coating agent for SeNPs synthesis due to the presence of hydroxyl side chain group [1].

Thus, the current study’s goal was to improve bacterial exopolysaccharide production through Taguchi statistical optimization design. Followed by utilizing it for coating selenium nanoparticles (AZEPS-SeNPs) to enhance their biological activity against pathogenic microbes, in vitro Adenocarcinoma cells in the human lung (A549) and diethyl nitrosamine (DEN)-induced lung cancer in vivo.

## Materials and methods

### Microorganisms

All the tested pathogens, namely: *Proteus vulgaris*, Methicillin-resistant *Staphylococcus aureus* (MRSA), *Staphylococcus aureus*,* Pseudomonas aeruginosa*,* Escherichia coli*,* Enterobacter aerogenes*, and *Candida albicans*, were kindly provided and identified using VITEK (BIOMERIEUX, USA) by the Pediatric Al-Shatby Hospital, Surveillance Microbiology Department at Alexandria. The isolated bacterial strain in the present work was identified as ***Azotobacter*** sp. genotypically using 16SrDNA sequencing.

#### Soil sampling

Different soil samples were collected from diverse fields in Rosetta, Al Buhayrah (Egypt) from the upper layer of the rhizospheric soil surrounding different crops namely, beans (*Vicia faba*), clover (*Trifolium*), wheat (*Triticum*), garlic (*Allium sativum*), and sweet potato (*Ipomoea batatas*), using aseptic plastic pouches and were transferred to the laboratory within an hour.

#### Medium used in Azotobacter isolation

Azotobacter medium agar used contained (g/L) glucose, 5.0; mannitol, 5.0; CaCl_2_.2H_2_O, 0.1; Na_2_MoO_4_.2H_2_O, 5.0; CaCO_3_, 5.0; KH_2_PO_4_, 0.1; K_2_HPO_4_, 0.9; MgSO_4_.7H_2_O, 0.1; FeSO_4_.7H_2_O, 0.01, and agar-agar, 20.0 at pH 7.3.

Soil samples (1 g) were serially diluted from 10^− 1^ to 10^− 7^, and 0.1 ml were then distributed over the freshly prepared *Azotobacter* agar media. The plates were incubated for 48 h (hrs.) at 30 ^o^C [12, 13]. *Azotobacter* isolates were visually examined and identified using Gram stain, spore formation, and biochemical reactions (catalase and oxidase tests) [14] and VITEK (BIOMERIEUX, USA).

#### Screening for *Azotobacter* Exopolysaccharide (AZEPS)-producing isolates

The production of EPS was determined through culturing the purified *Azotobacter* isolates on Congo Red Agar (CRA) media. The EPS production was detected by cell wall staining [15] and confirmed through the loop-touch approach.

#### Extraction and purification of exopolysaccharides

A pre-culture of *Azotobacter* sp. was prepared by using nutrient broth medium at 30 °C for 24 h. Then 1 ml of the freshly prepared inoculum (optical density (OD) 0.8) was added to 99 ml of *Azotobacter* broth medium and incubated for 24 h. at 30 °C under shaken conditions (100 rpm). The supernatant was collected by centrifugation for 20 min at 8,000 g, and 4 °C. Trichloroacetic acid (TCA) was slowly added to the supernatant for the removal of protein residues then recentrifuged at the same mentioned conditions. Cold absolute ethanol was added 2:1 (v/v) to the collected supernatant at 4ºC for 24 h. recentrifuged to extract the crude precipitated EPS (AZEPS) [16].

#### Exopolysaccharides antimicrobial activity

Antimicrobial activity of the crude EPS was established using the well diffusion method. 0.1 ml of the tested pathogens (1.5 × 10^6^ CFU/ml, 0.5 McFarland) were inoculated onto Muller-Hinton agar plates, then 25 µl of each purified water-soluble EPS suspension were loaded in all the wells, followed by incubation at 37 °C for 18 h. The inhibition zone diameters (mm) were recorded [17] at the end of the incubation period.

### EPS producer identification

The most potent isolate (with the highest antimicrobial activity) was identified using 16SrDNA sequencing that was amplified via the universal primer 27 F (5-AGAGTTTGATCMTGGCTCAG) and 1492R (5-TACCTTG TTACGACTT) [14], followed by a multiple sequence alignment using the National Center for Biotechnology Information (NCBI) database. The phylogenetic tree was constructed using the NT system and distance matrix analysis for the potent isolate.

### Composition of *Azotobacter* Exopolysaccharide (AZEPS)

#### Estimation of total sugar and protein content

Quantification of total sugar content was evaluated by the Phenol-sulfate acid method at 490 nm, utilizing D-glucose as a standard, while protein content was estimated using the Bradford method at 595 nm [18, 19].

### Characterization and identification of AZEPS

#### UV visible spectroscopy and Fourier transform infrared spectroscopy (FT-IR) analysis

A UV visible spectrophotometer (Shimadzu UV-1800) was employed to detect the absorption spectra at 200–800 nm of the extracted AZEPS.

Using the KBr technique, Fourier-transform infrared spectroscopy (Benchtop Cary 630 FTIR spectrometer, Agilent Technologies; Malaysia) was utilized to identify the functional and structural groups of the isolated AZEPS within the 400–4000 cm^-1^ spectrum.

##### Nuclear magnetic resonance spectroscopy analysis (NMR)

The extracted EPS was analyzed through nuclear magnetic resonance techniques (^13^C and ^1^H NMR) with the aid of a spectrometer (Bruker High Performance, Digital FT-NMR Spectrometer Avance III 400 MHz, Switzerland). Forty (40) mg of the extracted exopolysaccharide were disintegrated in deuterium oxide (D_2_O). The detected chemical shift was determined in parts per million (ppm) units [20].

##### Liquid chromatography Electrospray Ionization Tandem Mass Spectrometric (LC-ESI-MS)

The acquisition modes for positive and negative ions in ESI-MS were conducted using a Waters Corp. XEVO TQD triple-quadruple instrument (Milford, MA01757, USA). Acquit UPLC-BEH; C18 column (1.7 μm particle size, 2.1 × 50 mm) with gradient mobile phase elution was applied at a flow rate of 0.4 ml/min and two eluents (H_2_O acidified with 0.1% formic acid and acetonitrile acidified with 0.1% formic acid). Data acquisition Masslynx 4.1 software (Waters Corp., Milford, MA, USA) was utilized to control the process.

##### AZEPS Transmission electron microscopic (TEM) analysis

The analysis was evaluated using a transmission electron microscope (JEM-100 CX, Joel, USA), with a resolution of 3 nm and a voltage of 30 kV equipped with x-max50, an Oxford instrument EDX energy dispersive x-ray; the isolate cell morphology and ultrastructure were analyzed.

### Optimization of AZEPS production

#### Taguchi array design

The Taguchi orthogonal design, L27 (3^8^), was used to enhance the environmental and nutritional independent parameters to maximize the exopolysaccharide (Response R1) production. The L27 design was operated to explore 13 variables (X1- X13) at different levels (1, 2, 3) (Additional file 1: Table S1), where the number 27 denotes the number of experimental runs and L is the acronym for Latin square array (Table 1).

The S/N ratio, which refers to the relative measure of the desired objective to the variation from its mean, was estimated during data processing. The mean is the target value that represents the signal in the Taguchi design. On the other hand, the noise represents the response variable’s standard deviation. The larger is better quality characteristic was chosen for computing the S/N ratio, which was determined using the subsequent equation: (Eq. 1)


1


Where S/N is the signal-to-noise ratio, Y is the signal factor (EPS Production), and the symbol n stands for the experiment’s number of repeats.

**Table 1:** Coded design matrix for Taguchi orthogonal array

	Independent variables												
Trials	X1	X2	X3	X4	X5	X6	X7	X8	X9	X10	X11	X12	X13
1	1	1	1	1	1	1	1	1	1	1	1	1	1
2	1	1	1	1	2	2	2	2	2	2	2	2	2
3	1	1	1	1	3	3	3	3	3	3	3	3	3
4	1	2	2	2	1	1	1	2	2	2	3	3	3
5	1	2	2	2	2	2	2	3	3	3	1	1	1
6	1	2	2	2	3	3	3	1	1	1	2	2	2
7	1	3	3	3	1	1	1	3	3	3	2	2	2
8	1	3	3	3	2	2	2	1	1	1	3	3	3
9	1	3	3	3	3	3	3	2	2	2	1	1	1
10	2	1	2	3	1	2	3	1	2	3	1	2	3
11	2	1	2	3	2	3	1	2	3	1	2	3	1
12	2	1	2	3	3	1	2	3	1	2	3	1	2
13	2	2	3	1	1	2	3	2	3	1	3	1	2
14	2	2	3	1	2	3	1	3	1	2	1	2	3
15	2	2	3	1	3	1	2	1	2	3	2	3	1
16	2	3	1	2	1	2	3	3	1	2	2	3	1
17	2	3	1	2	2	3	1	1	2	3	3	1	2
18	2	3	1	2	3	1	2	2	3	1	1	2	3
19	3	1	3	2	1	3	2	1	3	2	1	3	2
20	3	1	3	2	2	1	3	2	1	3	2	1	3
21	3	1	3	2	3	2	1	3	2	1	3	2	1
22	3	2	1	3	1	3	2	2	1	3	3	2	1
23	3	2	1	3	2	1	3	3	2	1	1	3	2
24	3	2	1	3	3	2	1	1	3	2	2	1	3
25	3	3	2	1	1	3	2	3	2	1	2	1	3
26	3	3	2	1	2	1	3	1	3	2	3	2	1
27	3	3	2	1	3	2	1	2	1	3	1	3	2

### Selenium nanoparticles synthesis (AZEPS-SeNPs)

#### Preparation

The preparation of selenium nanoparticles (AZEPS-SeNPs) was formulated by adding 1 mg/ml of AZEPS solution to an equivalent amount of sodium selenite (Na_2_SeO_3_) (10 mM) as a stabilizing and reducing agent, the mixture was then Shaked for 30 min at 25 °C in dark container. A solution of 40 mM ascorbic acid (AsAc) was gradually mixed with the AZEPS-SeNPs combination over a period of 4 h. The color of the mixture transitioned from colorless to pale orange [21–23]. The AZEPS-SeNPs were subsequently collected via centrifugation at room temperature, followed by freeze-drying, and kept for further study.

#### Characterization

To assess the particle size (PS), zeta potential, and polydispersity index (PDI) of the produced nanoparticles, a dynamic light scattering (DLS) analysis was employed through the aid of a Malvern Zeta sizer. The UV-Vis spectroscopy was determined via a Shimadzu UV-1800 UV spectrophotometer at 25 °C in the spectrum range between 190 and 600 nm. The nanoparticles were also subjected to FTIR spectroscopy (Agilent Technologies; Benchtop Cary 630 FTIR spectrometer, Malaysia), which spanned a spectrum range of 400 to 4000 cm^− 1^. The produced AZEPS-SeNPs’ size, shape, and ultrastructure were studied using TEM (JEM-100 CX, 3 nm resolution at 30 kV, JOEL, USA) [24]. The AZEPS-SeNPs percentage of each elemental composition was measured via EDX (energy dispersive X-ray) spectroscopy (x-max50, manufactured by Oxford).

#### Antimicrobial activity of AZEPS-SeNPs

Well diffusion method and minimum inhibitory concentration (MIC) were used to evaluate the antimicrobial activity of the formulated AZEPS-SeNPs [25, 26]. All results were the mean of three trials.

### Anticancer and antioxidant activities in vitro

#### Antioxidant activity

DPPH free radical activity for AZEPS and AZEPS-SeNPs was carried out using an antioxidant assay kit (Colorimetric, K2078-100 abcam, BioVision Inc., USA). In 96-well plates, 2.5 ml of AZEPS and AZEPS-SeNPs with different concentrations were added to one ml methanolic DPPH solution. The solution was vigorously agitated, then stored at room temperature in the dark for 30 min, and then the absorption was measured at 595 nm [27]. An artificial antioxidant (Trolox) was used as an authentic sample.

The scavenging degree was calculated via the subsequent formula: (Eq. 2)

2 $$\eqalign{& {\rm{Scavenging effect }}\left({\rm{\% }} \right) = \cr & {{{\rm{control absorbance }} - {\rm{ sample absorbance}}} \over {control absorbance}} \times 100 \cr}$$

Where AZEPS is the sample absorbance and AZEPS-SeNPs is the solution absorbance. The control absorbance refers to the absorbance of the DPPH solution. The EC50, which represents the concentration at which 50% inhibition occurs, was determined by analyzing the graph depicting the scavenging effect percentages at various concentrations.

#### Cytotoxicity and cell culture assessment of AZEPS and AZEPS-SeNPs

Normal lung fibroblasts (WI38) and human lung cancer (A549) cell lines were provided by the American Type Culture Collection (ATCC). The cells were cultivated in DMEM (Invitrogen/Life Technologies) and enriched by 10 µg/ml of insulin (Sigma), 10% FBS (Hyclone, USA), and 1% penicillin-streptomycin. The MTT test was used to assess the viable cell count during 48 h at 37ºC in moistened air using 5% CO_2_. The cytotoxicity of AZEPS and AZEPS-SeNPs at varying concentrations (100, 25, 6.3, 1.6, and 0.4 µg/mL) was compared to Staurosporine (the reference drug) [28]. The absorbance was determined through employing a microplate reader (BDR-206, BIOLINE Diagnostic LLP, Delhi, India) at 450 nm. The IC50 values for AZEPS and AZEPS-SeNPs were determined [29]. Also, the selectivity index was determined according to (Eq. 3).

3 $$Selectivity~index=\frac{{IC50~of~normal~cells~}}{{IC50~of~cancer~cells}}$$

#### Cellular reactive oxygen species determination (ROS)

A549 and WI38 were added to varied doses of the produced AZEPS and AZEPS-SeNPs, and they were incubated for 72 h each. Total Reactive Oxygen Species (ROS) were determined in A549 and WI38 cells by using FITC channel flow cytometry (fluorescein isothiocyanate) via Assay Kit (Invitrogen, Thermo Fisher Scientific Inc., USA) for total ROS at 520 nm. The cellular amount of ROS was measured by combining 1 mL of cells with 100 µL of ROS assay stain solution and 1 mL of ROS assay buffer. The mixture was incubated for 1 h at 37 °C with 5% CO_2_. Following staining, cells can be preserved using IC Fixation Buffer (cat. 00-8222, USA), then maintained at a temperature range of 2–8 °C, shielded from light. They can then be analyzed using a ROBONIK P2000 flow cytometer (ELISA READER; India), using a wavelength of 450 nm.

#### Real-time (RT)–PCR analysis of the relative gene expression encoding pro- and anti-apoptotic markers (Caspase-3, BAX, and Bcl2)

RNAs were isolated from both the control group and the A549 cell line-treated group with AZEPS and AZEPS-SeNPs one at a time. Subsequently, complementary DNA (cDNA) was generated utilizing the Qiagen RNA extraction/Bio-Rad Syber Green PCR MMX kit (Bio-Rad Lab, Inc., Germany). Precise (Forward/Reverse) primers were used for each of the following genes: Caspase-3, BAX, Bcl2, and β-actin using software from the Rotor-Gen 6000 Series 1.7 (Build 87) (as presented in Table 2). The rule of 2^−∆∆CT^ was intended for relative gene expression estimation [30].

**Table 2:** Primers’ sequences of target genes

Tested gene	Primer sequence
Caspase-3	F: ATGTTTTCTGACGGCAACTTC
	R: AGTCCAATGTCCAGCCCAT
BAX	F: ATGTGTGTGGAGACCGTCAA
	R: GCCGTACAGTTCCACAAAGG
Bcl2	F: TGTTTGTGTGCTTCTGAGCC
	R: CACGCCATGTCATCATCAAC
β-actin	F: ATC GTG GGG CGC CCC AGG CAC
	R: CTC CTT AAT GTC ACG CAC GAT TTC

#### Cell cycle analysis assay

Apoptosis detection was assessed employing the ab139418 Propidium Iodide Flow Cytometry Kit, which is an Annex in the apoptosis detection kit V-FITC/PI (ab139418, USA) [31]. The A549 cells (1 × 10^6^) were collected and preserved in 66% ethanol at a temperature of 4 °C for a duration of 2 h. Subsequently, the cells were rehydrated using PBS and subjected to staining with propidium iodide and RNase for a period of 30 min. The data were analyzed on a flow cytometer using BD FAC SC Alibur, BD (Biosciences, Canada).

### In vivo trials

#### Animals

Forty albino male mice weighing 30–35 g (three-month-old) were obtained from the experimental animal house at the Pharmaceutical and Fermentation Industries Development Centre (PFIDC), City of Scientific Research and Technological Applications (SRTA-City), Egypt. All research animals were carefully reviewed by the Animal Ethics Committees (AEC), where ethical guidelines are issued. Animals’ treatment and experimental techniques were performed in accordance with the Institutional Animal Care and following the committee (IACUC) guidelines at Alexandria University with approval number AU04220924201. A 12-hour light-dark cycle was implemented to maintain the experimental mice at 23–25 °C and fed laboratory baseline chow and tap water for a 1-week adaptation phase. For induction of lung cancer, diethyl nitrosamine (DEN) (Sigma-Aldrich, USA) was utilized, which is considered a strong carcinogenic substance that induces the formation of tumors in various organs, including the lungs [32].

#### Experimental design

Following adaptation, the mice were subsequently allocated into four groups using a random selection method, with each group consisting of 10 mice.

Group 1 (Negative control): were injected intraperitoneally (IP) with 0.9% normal saline solution.

Group 2 (Positive control): were injected intraperitoneally with a 1% aqueous solution of DEN with a dose of 70 mg/kg once per week for two weeks, then combined with 375 µL injected subcutaneously of carbon tetrachloride (CCl_4_) dissolved in olive oil for an additional 6 weeks.

Group 3 (AZEPS treated): after tumor induction, 100 µL of AZEPS was applied orally/daily for 28 days.

Group 4 (AZEPS-SeNPs treated): after tumor induction, 100 µL of AZEPS-SeNPs was applied orally/daily for 28 days.

All mice were euthanized by the end of the experiment (28 days after tumor induction) to conduct biochemical and histopathological analyses. Blood was collected, and plasma was obtained by centrifuging the tubes’ contents for 10 min at 3000 rpm. An ice-cold lysis buffer was used to homogenize lung tissues to assess.

For histopathological study, lung tissues were maintained in a 10% buffered formalin solution [33], then stained by hematoxylin and eosin stain (H&E) to be examined by a light microscope.

#### Biochemical analyses

The tested biochemical parameters and hepatic function assessments, such as alanine aminotransferase (ALT) and aspartate aminotransferase (AST), were evaluated in plasma. Total protein (g/dl), total cholesterol, and total fat Triglyceride (mg/dl), albumin, total bilirubin (mg/dl), calcium (mg/dl), potassium (mg/dl), and creatine kinase-MB (CK-MB) (U/l) levels were assessed by using commercial kits following the manufacture instructions.

Malondialdehyde content was evaluated as thiobarbituric acid-reactive substances (TBARS), which is a lipid marker in homogenate tissues; the concentration was determined by utilizing the subsequent equation: (nmol/g wet tissue) = At × 0.156 × 10 [33, 34].

### Statistical analysis

The statistical calculations were performed and analyzed using Minitab19 software. In Taguchi design, during data processing, the signal-to-noise ratio and the target value (mean) were assessed using ANOVA at the 0.05 significance level.

## Results and discussion

### Isolation and phenotypic characterization of *Azotobacter* EPS producer

Rhizosphere samples from different farmlands around plants were screened for *Azotobacter* species isolation. The results of Azotobacter agar plates revealed that the isolated colonies were translucent, mucoid, and slimy with a distinctive ropiness. Ten isolates were selected and identified morphologically and biochemically. The isolated bacteria were Gram-negative bacilli with observed cysts and catalase and coagulase positive (Table S2) [35]. The presence of dormant cysts distinguishes the genus *Azotobacter* among the members of the *Azotobacteraceae* family [36].

Isolated Azotobacter species were examined for EPS production on Congo red agar. As shown in Fig. S1 black and dark red colonies were detected as a result of Congo red attachment to the cell wall [37].

#### Screening for exopolysaccharide production

The preliminary screening results in Table S2 showed the capability of ten isolates to produce extracellular polymeric substances (EPS) in filamentous form using *Azotobacter* broth media (Fig. S2). EPS was extracted from the selected isolates with a dry weight ranging from 0.23 to 2.35 g/L. The highest exopolysaccharide production was noticed with isolate AZ.6 (Fig. S31).

According to Núñez et al. [38], *Azotobacter vinelandii* produces large amounts of EPS during the growth period, wherever the EPS acts as a defensive barrier towards heavy metal toxicity. Aasfar et al. [39] revealed that *A. vinelandii* isolates were capable of synthesizing polysaccharides up to 5.26 g/L.

### Antimicrobial effect of the extracted AZEPS

The antibacterial activity of the isolated exopolysaccharides was examined by the well diffusion method. Preliminary screening showed that the EPS extracted from strain AZ.6 reported the highest proficiency against the examined pathogens (*Enterobacter aerogenes*,* K. pneumonia*,* Escherichia. coli*, and *Candida albicans* with 7, 7, 8 and 8 mm inhibition zone diameters, respectively) (Table S3).

The observed findings were in line with Patel et al. [40], who isolated EPS-producers from various soil samples that showed antibacterial action against *E. coli* with an inhibition zone diameter of 6 mm. EPS derived from marine bacteria was investigated for antibacterial effectiveness against human bacterial pathogens, including *Proteus vulgaris*, *Staphylococcus aureus*, and *Escherichia coli* with inhibition zones ranging between 7.1 and 9.9 mm [41].

### Identification of the most potent isolate

**Fig. 1:**
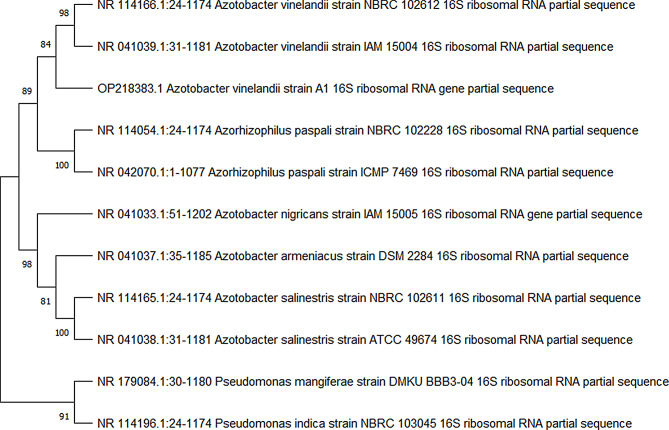
The phylogenetic tree of the selected isolate

In compliance with the NCBI database, the most potent isolate (coded AZ.6) was identified by 16 S rRNA sequencing and multiple sequence alignments. The phylogenetic tree of the promising strain was generated using a neighbor-joining tree using Mega 11 software, and it was revealed that the promising strain was *Azotobacter vinelandii* strain A1 with accession number OP218383 (Fig. 1).

#### Determination of total sugar and protein content of AZEPS

The crude *Azotobacter vinelandii* OP218383 exopolysaccharide protein content was evaluated using Bradford’s assay; the average concentration was 0.016 ± 0.55 mg/g, whereas the total carbohydrate content was 0.64254 mg/g.

### Characterization of the tested AZEPS

#### UV spectroscopy and FT-IR assessment

**Fig. 2:**
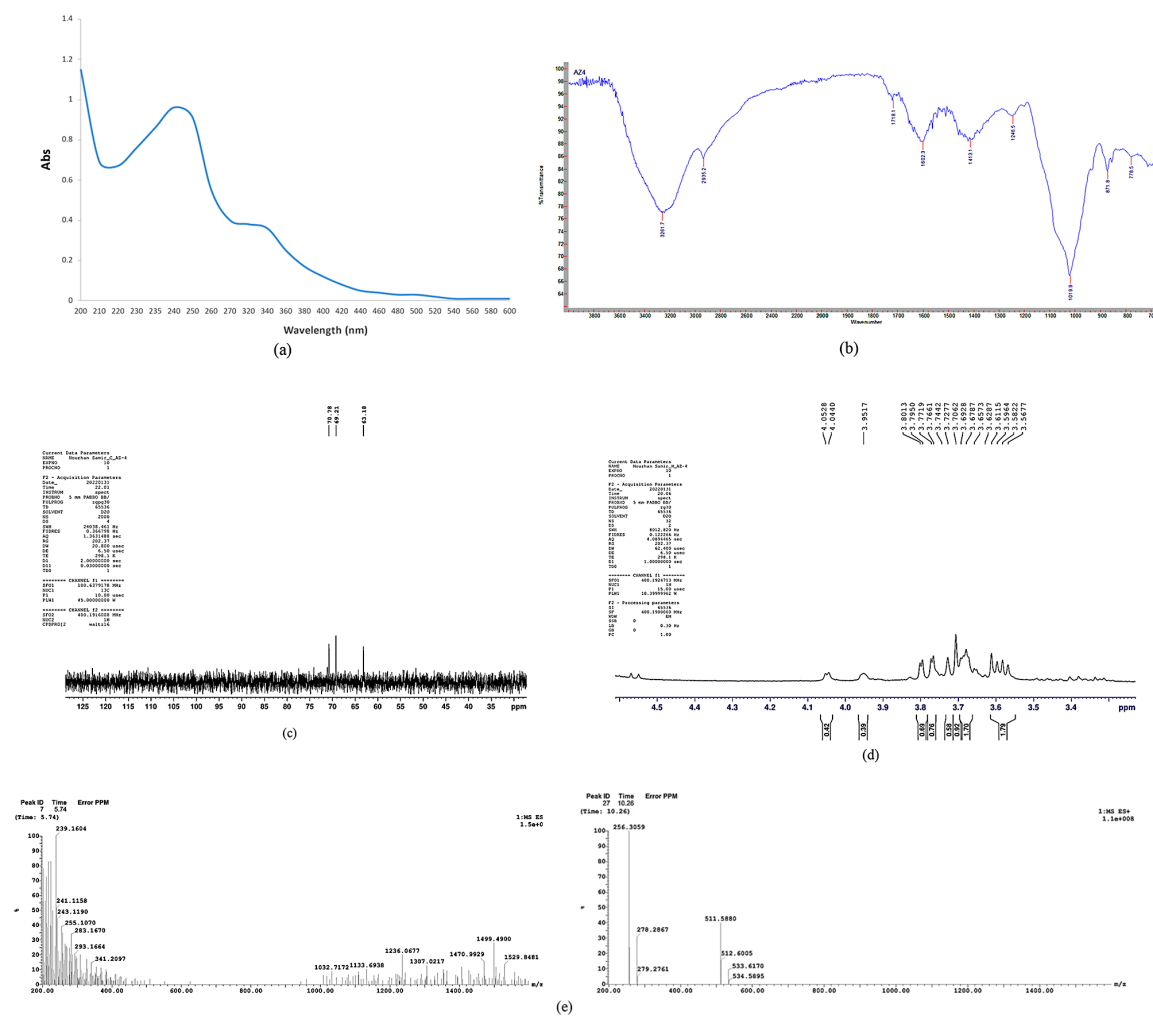
UV (**a**), FTIR (**b**), 1 H NMR (**c**) and ^13^C NMR (**d**) analysis, and EPS LC-ESI-MS analysis (**e**) of *Azotobacter vinelandii* OP218383 EPS

According to the UV spectrum, the absorption peak at approximately 240 nm indicated that the polysaccharides exhibited an O-polysaccharide glycosidic linkage (Fig. 2a) [19, 42].

FTIR analysis revealed a wide characteristic absorption peak around 3261.7 cm^− 1^, indicating the existence of hydroxyl groups (OH), which were responsible for the water solubility of the extracted exopolysaccharides, and NH groups, which were characteristic for the presence of polysaccharides (Fig. 2b). A weak absorption peak at 2935.2 cm^− 1^ showed C-H asymmetric stretching vibrations between carbon and hydrogen bonds in carbohydrates. In addition, the peaks at 1431.1 and 1602.3 cm^− 1^ were related to stretching vibrations of CO_2_ groups. A peak at 1781.1 cm^− 1^ signifies the existence of carbonyl groups. The absorption peaks near 1019.9 cm^− 1^ were considered critical evidence for the presence of CO-CC-OCH and β-pyran linkage. The peak at 871 cm^− 1^, indicates β-glycopyranosidic linkages [42].

#### NMR spectroscopy analysis

NMR is an effective approach to examining the bacterial polysaccharides’ structural characteristics. In order to interpret the structure of AZEPS produced from *Azotobacter vinelandii* (OP218383), ^1^H NMR and ^13^C NMR analyses were typically executed to characterize the structure of the glycosidic bond of AZEPS and further identify it [43]. Data suggested that the extracted exopolysaccharide was levan-type fructan.

The main resonance was shown in the anomeric region of ^1^HNMR (4.5–5.5 ppm). The signal found at 4.7 was assigned to anomeric hydrogens of β-linkage of glucose (1→3) (Fig. 2c), and signals in the range (3.5–4.05 ppm) were related to the fructose in levan [4]. Furthermore, levan was known to be composed of a repeating unit of fructose linked via β-(2–6)-glycosidic with several β-(2 − 1) branches. The β-(2–6) linkage, alternatively, has only been verified by the presence of a shifted signal at 63.35 ppm in the ^13^C NMR spectrum. While the detected signals at 69.21 and 70.78 ppm corresponded to the β -(2–6) linkage and indicated the presence of a (1–6) glycosidic bond (Fig. 2d) [4, 42].

#### LC-ESI-MS

The EPS was detected using LC-ESI-MS. The observable ions at 239.1, 341.2, 1133.6, 1307, 1470.9 m/z (Fig. 2e) exhibited a fructose signal matching to Fructooligosaccharides (FOS), confirming the existence of levan-type fructan [44]. Similarly, Mendonça et al. [20] reported that the peak m/z 511.2 revealed the presence of levan EPS produced by the *Paenibacillus* strain.

#### TEM analysis of *Azotobacter vinelandii* OP218383 cells

**Fig. 3:**
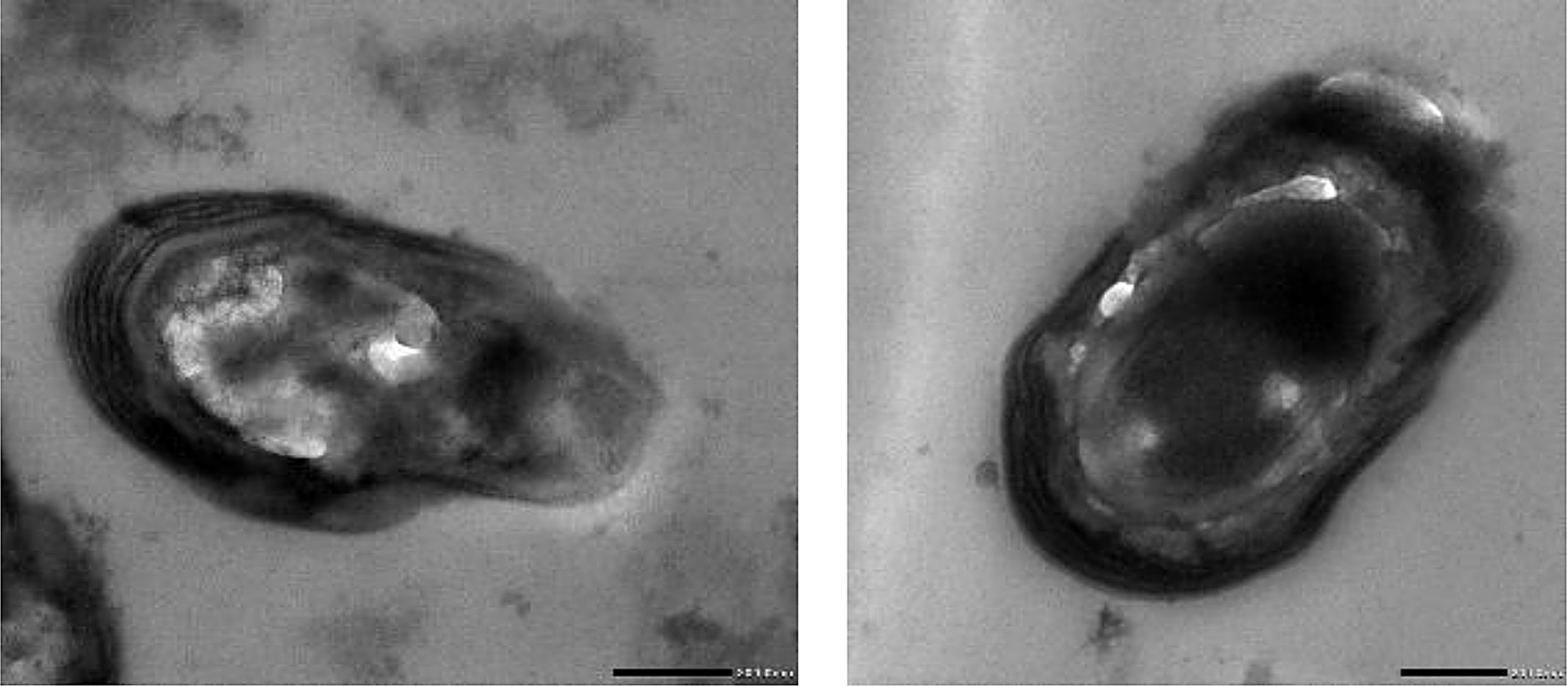
Azotobacter vinelandii OP218383 transmission electron microscopy

The existence of cysts in the resting stage distinguishes the genus Azotobacter from other members of the Azotobacteraceae family [36]. TEM morphological analysis of *Azotobacter vinelandii* OP218383 showed that the cells exhibited polymorphic cysts characterized by a condensed core of vegetative cells that included vacuoles and were surrounded by a bilayer shell as shown in Fig. 3.

### EPS production optimization

The Taguchi design was a useful statistical strategy for investigating the optimization of exopolysaccharide production involving several variables. The response values for the EPS production were analyzed via Mintab19 software. Analysis of variance (ANOVA) was used to verify statistical significance.

**Fig. 4:**
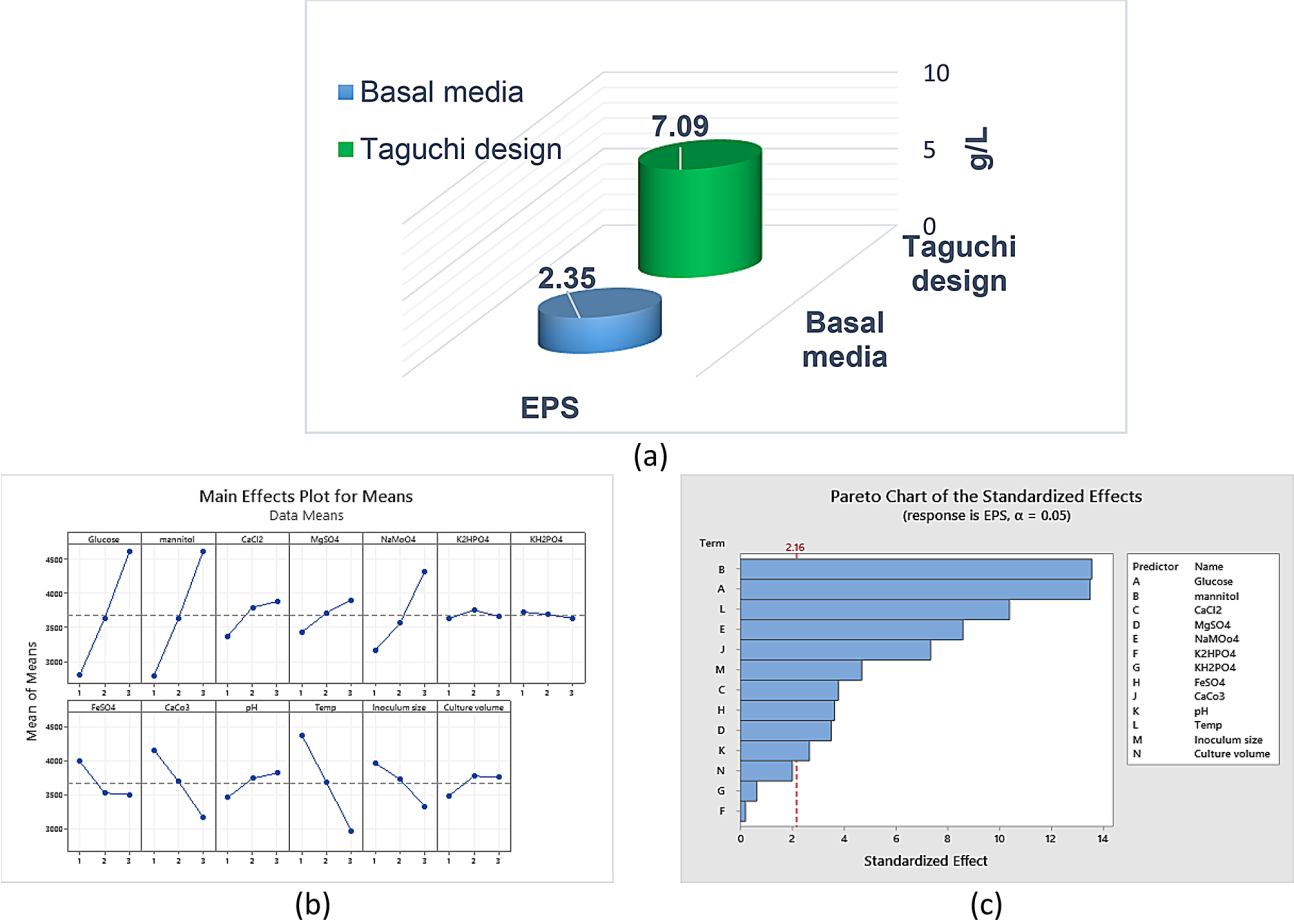
L-27 Taguchi designed a 3D graph for EPS production (**a**), a main effect plot of EPS (**b**), a pareto chart for the standardized effect of EPS (**c**)

The model’s F value shows that the responses were significant. The highest EPS production yield achieved was 7.09 g/l at trial no. 27, which represented a 3.017 folds’ increase compared to the baseline (Table 3; Fig. 4a). Data represented in Fig. 4b, Fig. S4a and Table S4 demonstrated the optimal values for each independent variable that were used to increase the EPS production.

ANOVA was performed to evaluate the findings of the preceding experiments and estimate the factors’ variation contributions to evaluate future interactions between different variables. Tables S5 and S6 showed that the *P* value was less than 0.05. As a result, the resulting equations (R1) were compatible with the experimental findings.

For EPS production, Mannitol concentration was reported as the most significant variable, followed by glucose and temperature, respectively (*P* < 0.05) (Fig. 4c). Additional file 1: Fig. 4b residual plot showed that the errors were uniformly distributed.

**Fig. 5:**
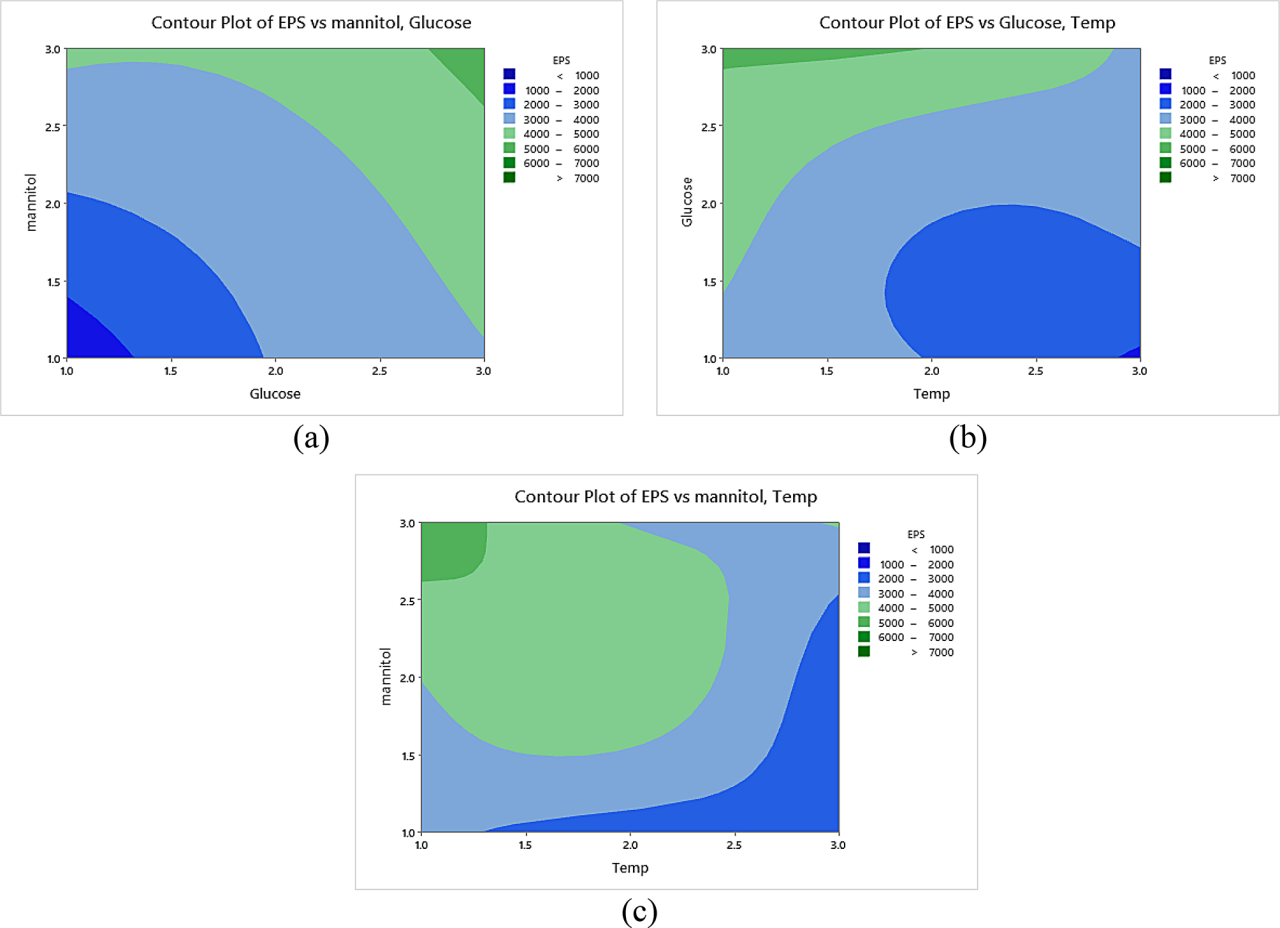
Contour plots for the correlation between: EPS versus mannitol and glucose (**a**), EPS versus glucose and temperature (**b**), EPS versus mannitol and temperature (**c**)

The statistical experimental analysis proved that the best combinations for EPS production were g/L: glucose, 30.0; mannitol, 30.0; CaCl_2_, 0.015; MgSO_4_, 0.010; NaMoO_4_, 7.0; K_2_HPO_4_, 1.0; KH_2_PO_4_, 0.5; FeSO_4_, 0.012; CaCo_3_, 3.0; pH, 8.0; temp, 25 ºC; inoculum size, 3.0 ml and culture volume, 50.0 ml. The contour plot interaction demonstrated the interaction between factors of the significant impacts on EPS production, as illustrated in Fig. 5 and Fig. S4c.

Raturi et al. [45] revealed that the proper selection of microorganisms and substrates is an essential step in high polyhydroxy butyrate (PHB) production. Several sub-optimal temperatures (8–25 ºC) were the best temperatures for enhancing EPS synthesis, most probably due to physiological stress induced by the lower temperature on bacterial cells [46].

**EPS regression analysis modeling**:

Mathematical models for dependent variable prediction were developed using linear regression analysis in Minitab 19.0 software.

4 $$\eqalign{{\bf{\it{R}}}1{\bf{\it{ }}}\left({{\bf{\it{EPS production}}}} \right) & = - 0.25 + 0.09072X1 + 0.09111X2 \cr & + 50.9X3 + 47.2X4 + 0.5772X5 \cr & + 0.028X6 - 0.136X7 - 121.9X8 - 0.2469X9 \cr & + 0.359X10 - 0.1397X11 - 0.3150X12 \cr & + 0.00540X13 \cr & ({{\bf{R}}^{\bf{2}}} = {\bf{98}}.{\bf{11}}) \cr}$$

**Table 3:** Optimization of *Azotobacter vinelandii* OP218383 EPS production using Taguchi orthogonal design

Runs	Glucose	mannitol	CaCl_2_	MgSO_4_	NaMoO_4_	K_2_HPO_4_	KH_2_PO_4_	FeSO_4_	CaCo_3_	pH	Temp	Inoculum size	Culture volume	Eps weight (g/L)
1	10.0	10.0	0.010	0.010	5.0	0.5	0.5	0.010	3.0	7.0	25.0	1.0	25.0	2.21
2	10.0	10.0	0.010	0.010	6.0	1.0	0.9	0.012	5.0	7.5	30.0	2.0	50.0	1.40
3	10.0	10.0	0.010	0.010	7.0	1.5	1.3	0.014	7.0	8.0	35.0	3.0	75.0	0.43
4	10.0	20.0	0.015	0.015	5.0	0.5	0.5	0.012	5.0	7.5	35.0	3.0	75.0	1.36
5	10.0	20.0	0.015	0.015	6.0	1.0	0.9	0.014	7.0	8.0	25.0	1.0	25.0	3.12
6	10.0	20.0	0.015	0.015	7.0	1.5	1.3	0.010	3.0	7.0	30.0	2.0	50.0	4.22
7	10.0	30.0	0.020	0.020	5.0	0.5	0.5	0.014	7.0	8.0	30.0	2.0	50.0	3.26
8	10.0	30.0	0.020	0.020	6.0	1.0	0.9	0.010	3.0	7.0	35.0	3.0	75.0	3.73
9	10.0	30.0	0.020	0.020	7.0	1.5	1.3	0.012	5.0	7.5	25.0	1.0	25.0	5.46
10	20.0	10.0	0.015	0.020	5.0	1.0	1.3	0.010	5.0	8.0	25.0	2.0	75.0	3.90
11	20.0	10.0	0.015	0.020	6.0	1.5	0.5	0.012	7.0	7.0	30.0	3.0	25.0	1.59
12	20.0	10.0	0.015	0.020	7.0	0.5	0.9	0.014	3.0	7.5	35.0	1.0	50.0	3.74
13	20.0	20.0	0.020	0.010	5.0	1.0	1.3	0.012	7.0	7.0	35.0	1.0	50.0	1.86
14	20.0	20.0	0.020	0.010	6.0	1.5	0.5	0.014	3.0	7.5	25.0	2.0	75.0	4.65
15	20.0	20.0	0.020	0.010	7.0	0.5	0.9	0.010	5.0	8.0	30.0	3.0	25.0	4.09
16	20.0	30.0	0.010	0.015	5.0	1.0	1.3	0.014	3.0	7.5	30.0	3.0	25.0	3.66
17	20.0	30.0	0.010	0.015	6.0	1.5	0.5	0.010	5.0	8.0	35.0	1.0	50.0	4.36
18	20.0	30.0	0.010	0.015	7.0	0.5	0.9	0.012	7.0	7.0	25.0	2.0	75.0	4.85
19	30.0	10.0	0.020	0.015	5.0	1.5	0.9	0.010	7.0	7.5	25.0	3.0	50.0	3.76
20	30.0	10.0	0.020	0.015	6.0	0.5	1.3	0.012	3.0	8.0	30.0	1.0	75.0	4.60
21	30.0	10.0	0.020	0.015	7.0	1.0	0.5	0.014	5.0	7.0	35.0	2.0	25.0	3.51
22	30.0	20.0	0.010	0.020	5.0	1.5	0.9	0.012	3.0	8.0	35.0	2.0	25.0	3.59
23	30.0	20.0	0.010	0.020	6.0	0.5	1.3	0.014	5.0	7.0	25.0	3.0	50.0	4.35
24	30.0	20.0	0.010	0.020	7.0	1.0	0.5	0.010	7.0	7.5	30.0	1.0	75.0	5.49
25	30.0	30.0	0.015	0.010	5.0	1.5	0.9	0.014	5.0	7.0	30.0	1.0	75.0	4.89
26	30.0	30.0	0.015	0.010	6.0	0.5	1.3	0.010	7.0	7.5	35.0	2.0	25.0	4.24
27	30.0	30.0	0.015	0.010	7.0	1.0	0.5	0.012	3.0	8.0	25.0	3.0	50.0	7.09

### Synthesis and characterization of selenium nanoparticles

**Fig. 6:**
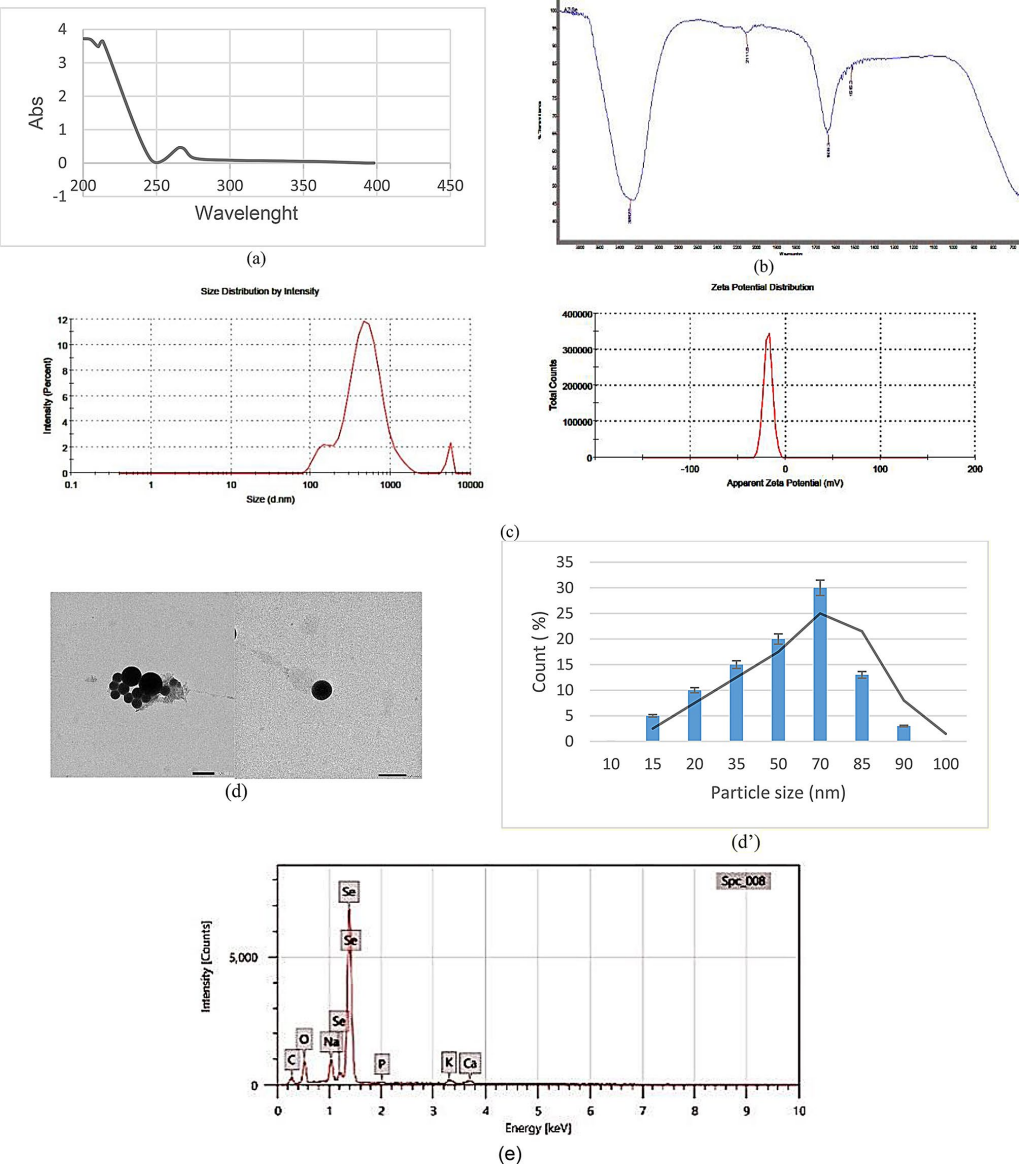
AZEPS-SeNPs physicochemical characterization; UV spectrum (**a**), FTIR (**b**), zeta potential, particle size (ps), and polydispersity index Zeta potential (**c**), TEM analysis (**d**, d’) and EDX (**e**)

*Azotobacter vinelandii* exopolysaccharide was used for the synthesis of AZEPS-SeNPs; bio-reduction of Na_2_SeO_3_ solution to Se^0^ element was confirmed by shifting the color of the reaction mixture to orange-red from colorless (Fig. S5), with maximal absorption between 240 and 340 nm and the characteristic peak was observed at 265 nm (Fig. 6a), in accordance with Hernández-Díaz et al. [47].

FTIR analysis for AZEPS-SeNPs was used to examine the functional groups responsible for nanoparticle formation and stability (Fig. 6b). The peak at 3282.5 cm^-1^ is highly significant, which indicates the presence of hydroxyl (-OH) groups of the aromatic rings’ groups responsible for the production of SeNPs, serve as a stabilizing and coating agent for selenium nanoparticles, which revealed the biopolymer association. 2111.6 cm^-1^ resembling C = C bond stretching (benzene), the peaks at 1636.3 cm^-1^ were related to the presence of the amide I band, C = O, stretch of the ester group, and N-H bending at 1515.3 cm^-1^. The detected results were consistent with Wang et al. [24], who synthesized stabilized selenium nanoparticles using *Corbicula fluminea* polysaccharide-protein complexes.

DLS is a non-invasive method that is used effectively for the determination of PDI and particle size (from submicron to 1 nm range) through measuring the hydrodynamic diameter of nanomaterials that undergo Brownian movement in solution over a short period at low cost [48]. The size distribution of AZEPS-SeNPs was recorded by zeta potential and PDI (-18.5 mv and 0.384, respectively) (Fig. 6c). Analysis of size distribution by TEM is based on the number of the particles over a measured size area [49], as shown in (Fig. 6d’). TEM study of AZEPS-SeNPs revealed that the particle size ranged from 65 to 70 nm, spherical-shaped and uniformed (Fig. 6d). Similarly, Wang et al. [24] synthesized polysaccharide–protein complexes (PSP-SeNPs) with a size range of 40 and 70 nm. Also, Zhang et al. [50] synthesized selenium nanoparticles decorated by *Spirulina platensis* polysaccharide (with a size range of 73.42 ± 0.69 nm).

The examination of the synthesized selenium nanoparticles (SeNPs) using EDX declared that the Se atom percentage was 24.49 ± 0.14%. The absorption peak characteristic for selenium was detected at 1.39 keV. While oxygen and carbon atom percentages were 28.24 ± 0.38%, and 39.85 ± 0.59% respectively (Fig. 6e), which resulted from the coating exopolysaccharides [47, 50].

### Antimicrobial activity of AZEPS-SeNPs

AZEPS-SeNPs showed the highest antibacterial efficacy against *Staphylococcus aureus*, with a zone of inhibition diameter reaching 36.6 mm, followed by *E. aerogenes*, *E. coli*, *Klebsiella pneumoniae*, and MRSA with inhibition zone diameters of 31.6, 29.7, 29.6 and 28.4 mm, respectively (Fig. S6). El-Zayat et al. [51] reported similar results of selenium nanoparticles synthesized greenly via *Ephedra aphylla* aqueous extract, which demonstrated effective antibacterial efficacy against *E. coli*, *Staphylococcus aureus*, *Klebsiella pneumoniae*, and *Candida albicans*.

AZEPS-SeNPs reported bactericidal activity against the tested pathogens with MIC index > 4 (Table 4). Ferro et al. [52] stated that SeNPs showed antibacterial activities against *E. coli* and MRSA due to bacterial cellular ROS levels’ boosting and inducing bacterial cell lysis. SeNPs can induce rupture in the cell wall and inhibit the protein and DNA syntheses [47].

**Table 4:** AZEPS and AZEPS-SeNPs antimicrobial activity, MIC, MBC, and MIC index

Tested pathogen	AZEPS				Na_2_SeO_3_				AZEPS-SeNPs			
	Inhibition Zone IZ (mm)	MIC (µg/mL)	MBC (µg/mL)	MIC index	Inhibition Zone IZ (mm)	MIC (µg/mL)	MBC (µg/mL)	MIC index	Inhibition Zone IZ (mm)	MIC (µg/mL)	MBC (µg/mL)	MIC index
*E. coli*	8.0	32	128	4	12.1	32	128	4	29.7	4	32	8
*Ent. aerogenes*	7.0	32	128	4	18.5	16	64	4	31.6	8	64	8
*Staphylococcus aureus*	6.0	64	128	2	17.4	16	64	4	36.6	4	32	8
*Klebsiella pneumoniae*	7.0	64	256	4	15	32	128	4	29.6	4	32	8
*P. vulgaris*	R	256	256	1	R	256	256	1	12.3	16	128	8
*MRSA*	5.5	64	128	2	R	256	256	1	28.4	8	64	8
*Candida albicans*	8.0	16	64	4	16.9	16	128	8	49.3	4	32	8

MIC: minimum inhibitory concentration; MBC: minimum bactericidal concentration

### AZEPS and AZEPS-SeNPs antioxidants and cytotoxic effects

#### Antioxidant effect

Li et al. [28] stated that during the last few decades, the utilization of natural polysaccharides has been increasing attention due to minimal toxicity, biocompatibility, biodegradability, protectiveness, stability, and targeting capability. They also exhibit antioxidant and anti-tumor activity. However, SeNPs have garnered increasing attention as a promising method for cancer treatment. Despite SeNPs great biological activity and antioxidant properties, their application has been limited due to their poor solubility, stability, and dispersal characteristics. Fortunately, polysaccharides exhibit the ability to effectively encapsulate and stabilize Se nanoparticles, which enhances the stability and anticancer properties of SeNPs. Also, the SeNPs concentration increment improved their antioxidant activity [26]. This was demonstrated by the present results using varied concentrations from 2.5 to 100 g/mL. AZEPS and AZEPS-SeNPs demonstrated a concentration-dependent DPPH free radical scavenging action (Table 5). The results demonstrated that Trolox (positive control) had the highest antioxidant capacity, followed by AZEPS-SeNPs and AZEPS. At 100 g/mL, as a result of the senergetic effect of the combination of AZEPS with SeNPs, the DPPH scavenging efficiency of AZEPS-SeNPs exceeds the AZEPS and positive control percentages (96.8, 90.2, and 92.9%, respectively), demonstrating a greater activity of the synthesized nanoparticles.

In harmony with Chen et al. [53] who reported that *Polygonatum sibiricum* polysaccharide (PSP) exhibits lower DPPH radical scavenging than SeNPs; however, the combination of PSP and SeNPs demonstrated a stronger scavenging potential than SeNPs.

According to Ferro et al. [52], pectin-stabilized selenium nanoparticles (PEC-SeNPs) displayed increased antioxidant activity in both Trolox equivalent antioxidant capacity (TEAC) experiments and DPPH radical scavenging.

**Table 5:** AZEPS, AZEPS-SeNPs, and Trolox DPPH scavenging activity

Conc (µg/mL)	AZEPS	AZEPS-SeNPs	Trolox (positive control)
100	90.2	96.8	92.9
50	66.1	80	84.1
10	39.5	52	62.8
5	10.6	14.9	27.7
2.5	0	0	0
EC50	20.79 ± 1.2c	14.54 ± 0.82b	8.241 ± 0.46a

Different letters a, b and c within the same column indicate that they are significantly different at *p* < 0.05 (letter a is the smallest, followed by b, and finally the letter c is the highest one)

#### Cytotoxic properties

Regardless of whether chemotherapy is more successful in treating lung cancer, it is less preferred due to its severe adverse effects on normal cells. Although polysaccharides showed limited anticancer efficiency in A549 cells, Wang et al. [54] stated that one of the most effective ways to improve the polysaccharides’ anti-cancer properties is to combine them with selenium nanoparticles. Also, it was reported that SeNPs showed a highly potential cytotoxic effect on A549 cells, with an IC50 of 27.10 µg/ml; however, when mixed with polysaccharide, the IC50 value decreased to 13.59 µg/ml, resulting in a maximal inhibition rate of 70.3%.

**Fig. 7:**
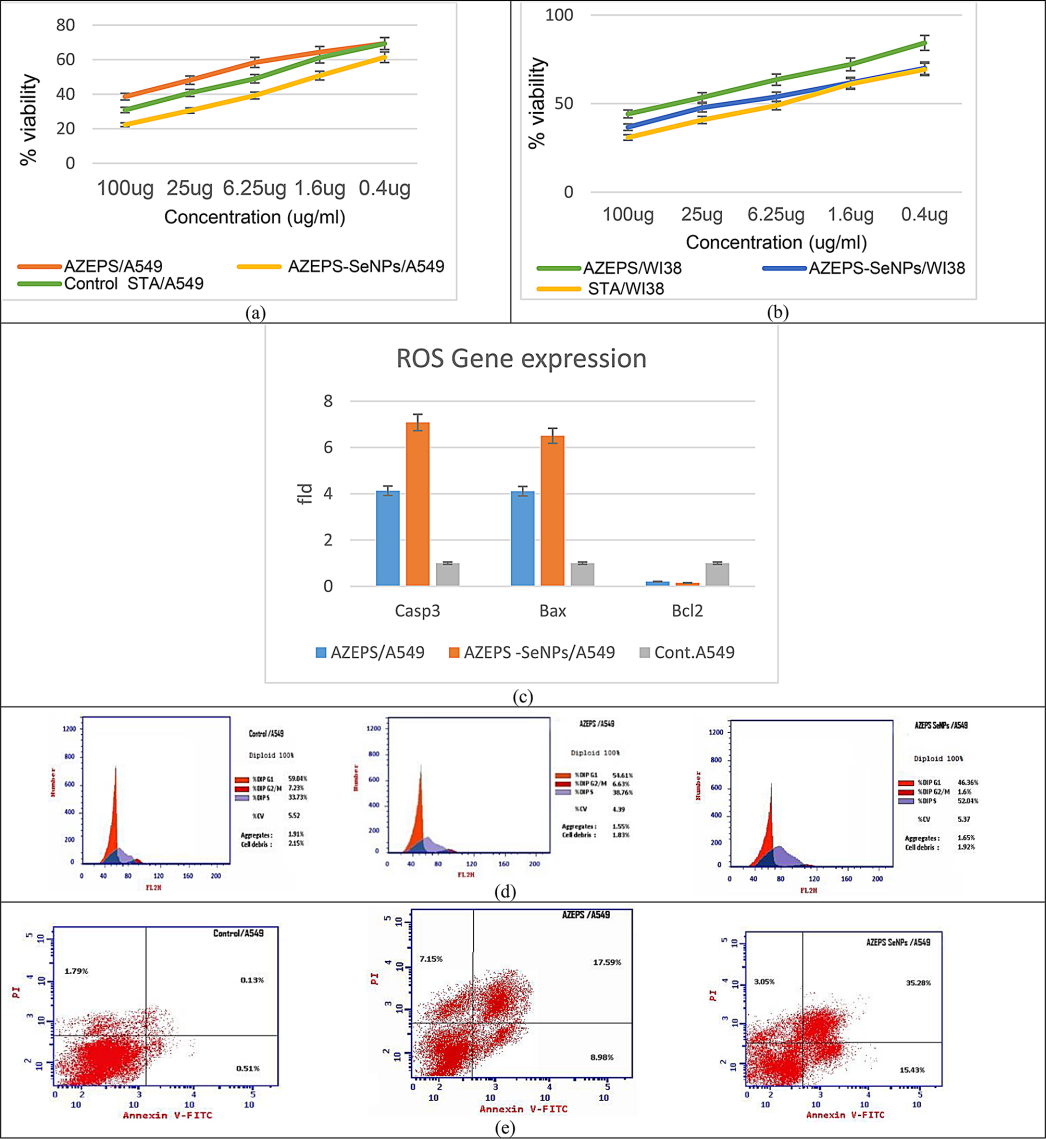
Cell viability of A549 (**a**) and WI38 (**b**) as affected by Staurosporine, AZEPS, and AZEPS-SeNPs concentrations; expression of Casp3, Bax, and Bcl-2 using RT-PCR (**c**); The flow cytometric profile of control and AZEPS-SeNP-treated A549 cells (**d**); Annexin apoptosis assay for A549 cells, treated cells via V-FITC/PI double staining technique (**e**)

AZEPS and AZEPS-SeNPs cytotoxic effects, as opposed to normal WI38 cells, were evaluated and compared to Staurosporine. While the IC50 against A549 cells was estimated to analyze the anti-lung cancer effects, which was 17.49 ± 0.85 for AZEPS, and when combined with SeNPs (AZEPS-SeNPs), the IC50 reached 1.724 ± 0.08, revealing the synergetic effect by 3.7 folds (Table S7). According to the calculated therapeutic index (IC50 WI38/ IC50 A549), both AZEPS-SeNPs and AZEPS were (7.18 ± 0.21 and 2.37 ± 0.11, respectively) considered completely safe. The viability effect of AZEPS and AZEPS-SeNPs on A549 and WI38 cells was dose-dependent, with concentrations varied from 0.4 to 100 g/mL (Fig. 7a-b).

According to Sachin & Karn [55], biosynthesized SeNPs revealed excellent cytotoxic effects when assessed against the human breast cancer cell line (MCF-7) and the A549 cell line. SeNPs showed minimal cytotoxicity compared to selenium compounds, revealing remarkable anticancer, therapeutic characteristics, and stability due to the presence of EPS. Cao et al. [56] stated that polysaccharides from *Grateloupia Livida* coating selenium nanoparticles (GLP-SeNPs) have revealed significant antioxidant activity and targeted cytotoxicity toward several human cancer cells, particularly A549 cells. Zhou et al. [57] indicated that polysaccharide-based selenium nanoparticles produced by *Chaenomeles speciosa* (CSP-SeNP3) have shown a remarkable cytotoxic effect against MCF-7, HepG2, and A549 cancer cells, indicating a prospective application in cancer treatment.

#### Determination of genetic expressions and impact on ROS of AZEPS and AZEPS-SeNPs

Results showed that AZEPS-SeNPs and AZEPS-producing ROS in cancer cells were moderately increased (1.33, 1.17 and 1 respectively), as shown in Table 6. According to Nakamura & Takada [58], SeNPs were correlated with increased ROS generation besides increased anti-proliferation efficacy against cancer. SeNPs were diffused and depleted malignant cells via several molecular mechanisms, including activation of the intrinsic apoptosis pathway [52].

The present investigation revealed that cell death appeared to be associated with several caspases’ activation. Activated caspase-9/3 was regarded as a critical operator for the transmission of apoptotic signaling [59]. According to RT-PCR results (Fig. 7c), AZEPS and AZEPS-SeNPs have proapoptotic potential for the generation of Bax and caspase 3. Caspase 3 expression increased by 4.13 and 7.08-fold when treated with AZEPS and AZEPS-SeNPs in A549 cells, respectively. Furthermore, in AZEPS- and AZEPS-SeNPs-treated A549 cells, the expression of Bax was elevated by 4.111 and 6.505-fold, respectively, while the anti-apoptotic gene Bcl2’s was lowered to 0.205 and 0.151-fold, respectively. In agreement with Lin et al.’s [59] findings, who reported that *Hedyotis diffusa* polysaccharide (HDP) stimulated the caspase-9/-3 apoptotic cascade and caused apoptosis in A549 cells by stimulating the apoptotic cascade of caspase-9/-3. Selenium nanoparticles coated with laminarin polysaccharides (LP-SeNPs) increased Bax expression while decreasing Bcl-2 expression in HepG2 cells. Bax resulted in the activation and production of several apoptotic genes, including cytochrome c, and the activation of caspase 9 as an overexpression of proapoptotic. The upregulation of Bax/cleaved caspase 9 and downregulation of Bcl-2 indicate the involvement of the mitochondrial pathway in apoptosis [60, 61].

**Table 6:** AZEPS and AZEPS-SeNPs induce ROS production in the A549 cell line

Sample	ROS	
	∆RFU	Fold increase
AZEPS /A549	167,416	1.17 ± 0.05b
AZEPS-SeNPs/A549	191,016	1.33 ± 0.07b
H_2_O_2_/A549	218,125	1.52 ± 0.06c
Cont.A549	142,733	1 ± 0.01a

∆RFU, relative fluorescence unit

#### Cell cycle assay

**Fig. 8:**
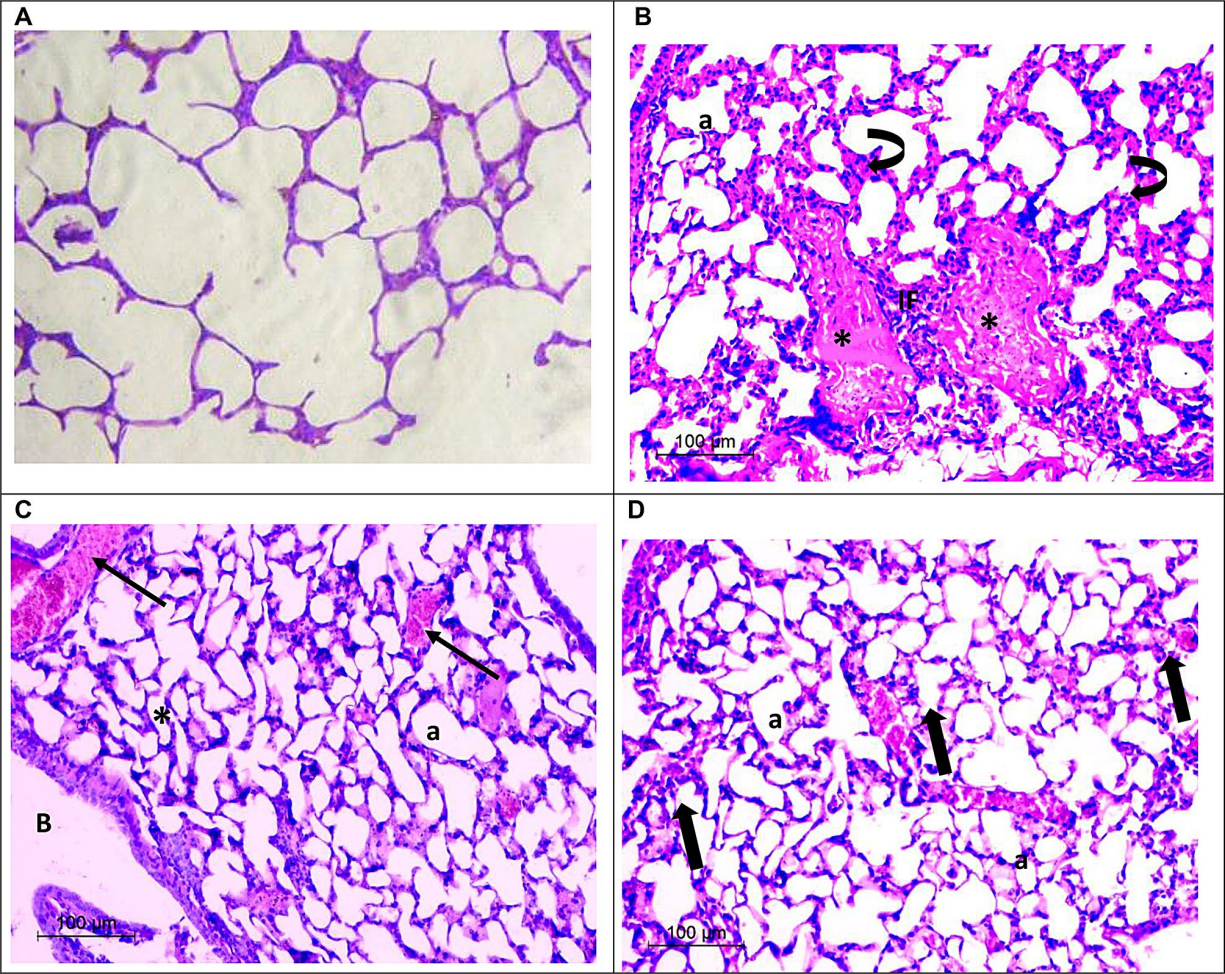
Light micrograph demonstrating H&E-stained lung tissue sections, (**A**): negative control group of lung tissue. (**B**): a positive control group of lung tissue with adenocarcinoma showing severe mononuclear cell infiltration (IF), thickening of the interalveolar septa (curved arrows), alveoli congested with blood (*), and alveoli (**a**). (**C**): illustrating lung tissue after AZEPS showing collapsed alveoli (*), some alveoli congested with blood (arrows), bronchus with disorganized epithelium (**B**) and alveoli (**a**). (**D**): demonstrating lung tissue after AZEPS-SeNPs showing relatively normal parenchyma, mild focal proliferation of the interalveolar septa (thick arrows), and near-to-normal organized alveoli (**a**)

The prevalence of the cell cycle in A549 cells caused by AZEPS and AZEPS-SeNPs was analyzed by flow cytometry. After treatment with AZEPS and AZEPS-SeNPs, the assay results exhibited A549 cell apoptosis, which was notably increased in early, late apoptosis, and necrosis leading to death relative to the control group ((33.72 and 7.15) and (53.76 and 3.05)), respectively (Table S8 and Fig. 7d-e). The suppression of cell proliferation is linked to the interruption of the cellular division process. The A549 cell cycle distribution was examined using AZEPS and AZEPS-SeNPs to establish a correlation between cell cycle arrest and the inhibitory effects of AZEPS and AZEPS-SeNPs on cell growth. Results revealed that AZEPS and AZEPS-SeNPs trigger the arrest of cell growth in the S phase (Table S9). The results were compatible with that of Cao et al. [56], who detected that the anti-proliferative activity of GLP-SeNPs against A549 was principally induced at S-phase cell cycle arrest. The results resemble those of Zhou et al. [57] (See Fig. 8).

### In vivo anti-lung cancer effect of AZEPS and AZEPS-SeNPs

#### Impact of AZEPS and AZEPS-SeNPs on biochemical parameters: protective and therapeutic effects

Examination of lipid profile, liver function, renal function, hematological parameters, tumor indicators, and stress were measured in the serum of tumor-induced and treated animals. Tumors may potentially lead to chronic kidney disorders. The identification of cancer progression contributed to kidney function abnormalities, which were detected through elevated levels of urea, creatinine, and hyperuricemia. Lipid peroxidation and cancer metastasis are associated with increased levels of TBARS (a harmful byproduct), which may be considered a signal damage to lung tissue and other cellular structures [62]. Selenium nanoparticles exhibit strong antitumor activity, which is an effective strategy for the improvement of the antitumor activity of polysaccharides and accordingly has great potential in the field of cancer treatment. The combination of polysaccharide with selenium (PLP-SeNPs) significantly improved the anti-tumor activity and effectively blocked tumor proliferation and metastasis by inhibiting angiogenesis in vivo [54]. As shown in Table 7, Group 2 (the positive control) showed a low level of total protein, albumin, and calcium that was associated with a high level of lipid profile (total cholesterol, triglyceride). Parameters related to liver function (total bilirubin, ALT, AST, and CK-MB), functional kidney parameters (urea, creatinine, and uric acid), and prooxidant levels at *P* < 0.05 in comparison with the negative control group. Alternatively, AZEPS-SeNPs (Group 4) treatment was better than AZEPS (Group 3) at *P* < 0.05, although AZEPS and AZEPS-SeNPs improved all tested parameters but failed to normalize them.

In agreement with the present study, Haddar [63] stated that DEN/CCl_4_ induction led to high lipidemia, kidney failure, and liver cancer and motivated the production of oxidative stress, while the polysaccharide showed as a hepatic and kidney protective agent. However, AZEPS-SeNPs and AZEPS-treated groups significantly reduced the assessed parameters.

**Table 7:** Protective and therapeutic effects of AZEPS-SeNPs and AZEPS on biochemical parameters

Biochemical parameters	Group − 1 (Negative control)	Group − 2 (positive control-DEN)	Group − 3 (DEN + AZEPS)	Group − 4 (DEN + AZEPS-SeNPs)
Total protein (g/dl)	63.45 ± 0.18d	46.1 ± 0.24a	52 ± 0.14b	59.1 ± 0.12c
Total Cholesterol	82.2 ± 0.12a	115 ± 0.7d	100 ± 0.23c	91 ± 0.18b
Triglyceride (mg/dl)	64.4 ± 0.49a	95.4 ± 0.19d	89 ± 0.25c	72.1 ± 0.10b
Albumin (g/dl)	3.7 ± 0.07d	2.84 ± 0.06a	3.11 ± 0.016b	3.25 ± 0.01c
Total bilirubin (mg/dl)	0.28 ± 0.01a	1.69 ± 0.02d	0.76 ± 0.01c	0.42 ± 0.00b
ALT (U/L)	22.54 ± 0.08a	113 ± 1.15d	63 ± 1.05c	27.66 ± 0.16b
AST (U/L)	150 ± 2.86a	285 ± 0.66c	208 ± 1.24b	159.5 ± 1.64a
Urea (mg/dl)	35.67 ± 0.13a	52.3 ± 0.94d	44.2 ± 0.39c	39 ± 0.45b
Creatinine (mg/dl)	0.34 ± 0.01a	0.65 ± 0.008d	0.59 ± 0.014c	0.49 ± 0.014b
Uric acid (mg/dl)	1.35 ± 0.008a	3.44 ± 0.04d	3.01 ± 0.01c	2.69 ± 0.02b
Ca (mg/dl)	7.61 ± 0.14d	6.29 ± 0.05a	6.67 ± 0.06b	6.9 ± 0.093c
K (mmol/L)	5.39 ± 0.07a	5.11 ± 0.01a	5.16 ± 0.01a	5.23 ± 0.02a
CK-MB (U/L)	0.31 ± 0.006a	0.75 ± 0.01d	0.53 ± 0.01c	0.42 ± 0.008b
TBARS (nmol/mg)	4.1 ± 0.15a	10.1 ± 0.09d	6.9 ± 0.06c	5.4 ± 0.06b

mmol/L, millimoles per liter/ nmol/mg, nanomole per milligrams / U/L, Units Per Liter / mg/dl, milligrams per deciliter/ g/dl, grams per deciliter

#### Histopathological examination of lung

Lung histological sections of the negative control group showed normal alveolar architecture with thin and well-organized alveolar space (Fig. 8a). Lung histological sections of the positive control group showed severe inflammation in the lung parenchyma and adventitia because of the carcinogen DEN/CCl4 administration (Fig. 8b). The mononuclear cell infiltration can be seen within the thick interalveolar septa indicating inflammation. It can be noticed that some alveoli were congested with blood. On the other hand, histological study results after AZEPS treatment showed collapsed alveoli (Fig. 8c), moreover, the bronchus showed disorganized epithelium. Although micrographs appeared relatively normal after applying AZEPS-SeNPs with mild focal proliferation of the interalveolar septa and near to normal organized alveoli (Fig. 8d).

## Conclusion

The present investigation revealed that:

- AZEPS was proved to be composed of Levan-type fructan with a protein content of 0.016 ± 0.55 mg/g and a sugar content of 0.64254 mg/g.
- Optimization of the environmental and nutritional parameters using Taguchi experimental design maximized the EPS production.
- The optimized EPS (AZEPS) was used to encapsulate and stabilize selenium nanoparticles (AZEPS-SeNPs) and enhance its antimicrobial activity against *Staphylococcus aureus* and *Enterobacter aerogenes*.
- AZEPS-SeNPs showed a bactericidal effect against the tested pathogens.
- *Azotobacter vinelandii* polysaccharides enhanced SeNPs antioxidant, stability, and anticancer properties.
- AZEPS-SeNPs were considered completely safe.
- AZEPS-SeNPs have the proapoptotic potential (through Bax and caspase 3 generations) and anti-apoptotic effect through inhibiting Bcl2’s gene expression.
- Moreover, the synthesized AZEPS-SeNPs showed potent anti-lung cancer effects in an animal model.

## Electronic supplementary material

Below is the link to the electronic supplementary material.

Supplementary Material 1

## Data Availability

Data supporting this study will be available from the National Center for Biotechnology Information (NCBI) database. https://www.ncbi.nlm.nih.gov/search/all/?term=OP218383 (Accessed on 19 August 2022).
