# Supplementary material for: In vitro and in vivo studies of selenium nanoparticles coated bacterial polysaccharide as anti-lung cancer agents
Source: Microb Cell Fact. 2024 Dec 19;23:339. doi: 10.1186/s12934-024-02601-z (PMC11658177; doi:10.1186/s12934-024-02601-z)
Supplement: Supplementary file 1 — Supplementary Material 1 [file 12934_2024_2601_MOESM1_ESM.docx]

**Additional information**

**Assessments of anticancer effect of selenium nanoparticles coated bacterial polysaccharide**

**Nourhan S. Shehata**^1,2*^**, Bassma H. Elwakil**^1^ **, Salma S. Elshewemi**^3^**, Doaa A. Ghareeb**^4^**, Zakia A. Olama**^2^

^1^Department of Medical Laboratory Technology, Faculty of Applied Health Sciences Technology, Pharos University in Alexandria, Alexandria, Egypt

^2^Department of Botany and Microbiology, Faculty of Science, Alexandria University, Egypt

^3^Zoology Department, Faculty of Science, Alexandria University, Egypt

^4^Bio-Screening and Preclinical Trial Lab, Biochemistry Department, Faculty of Science, Alexandria University, Alexandria 21526, Egypt

^*^ Corresponding author: **Nourhan S. Shehata**, Department of Medical Laboratory Technology, Faculty of Applied Health Sciences Technology, Pharos University in Alexandria, Alexandria, Egypt., email: [nourhan.shehata@pua.edu.eg](mailto:nourhan.shehata@pua.edu.eg) Tel: +0201276721783

| **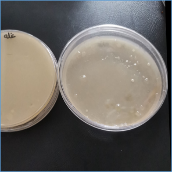** | | **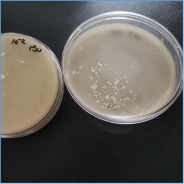(a)** | (b)**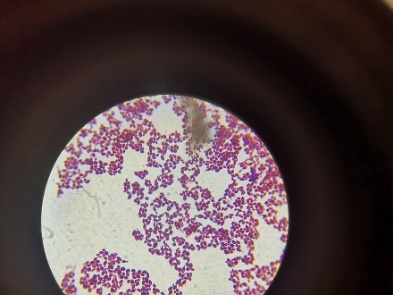** |
| --- | --- | --- | --- |
| **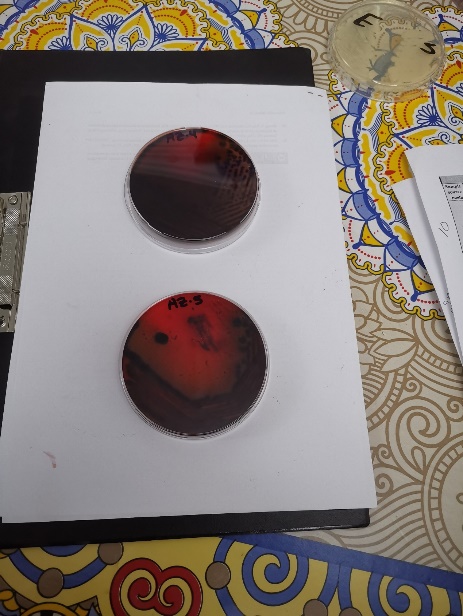(c)** | **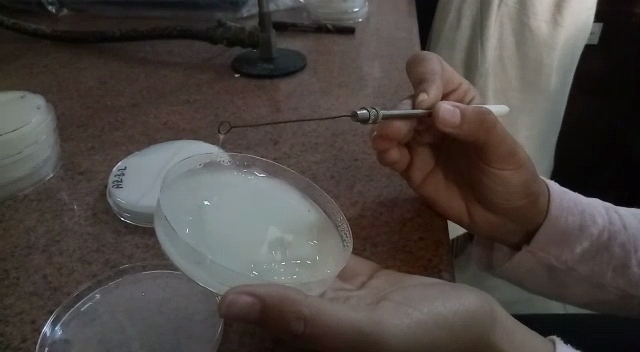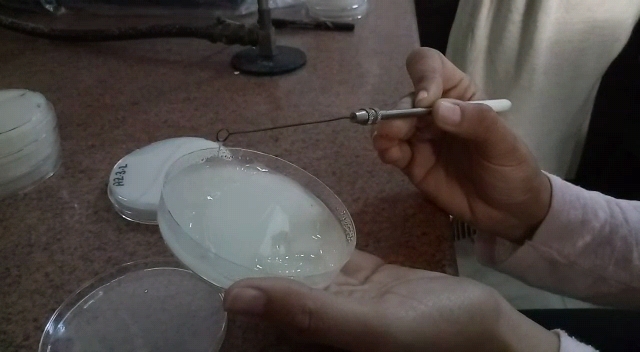(d)** | | |
| Fig. S1. Isolation of Azotobacter species from different samples, morphological identification using Gram stain(b), examination of Azotobacter species for EPS production on Congo red agar(c), ropiness of isolated colonies (d). | | | |

| **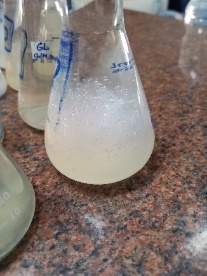** |
| --- |
| Fig. S2. Production of exopolysaccharide (EPS) in the form of filaments in Azotobacter broth media. |

Fig.S3: EPS-producing bacteria.

| 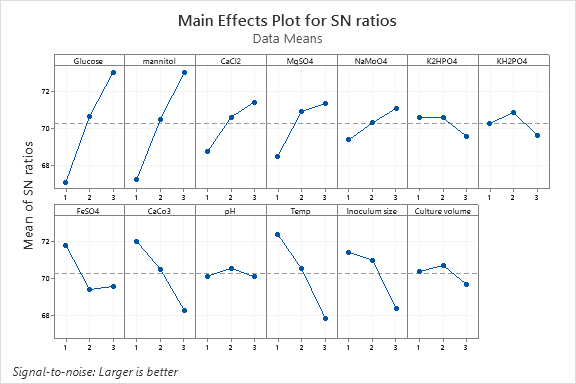(a) |
| --- |
| 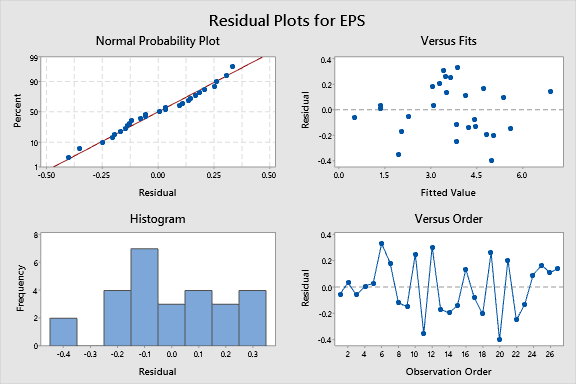(b) |
| 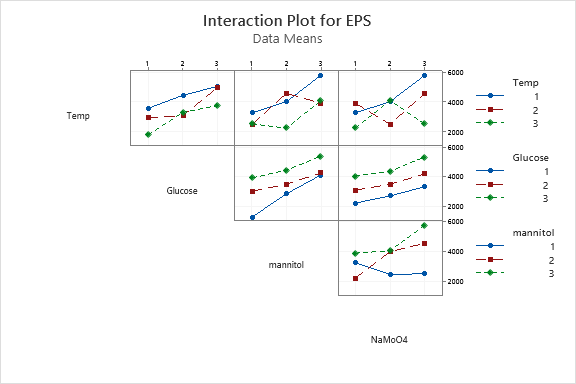(c) |
| Fig. S4 S/N ratios for relative factors affecting EPS(a), Residual Plot for EPS (b), interaction plot between EPS and the most effective parameters (c). |

| 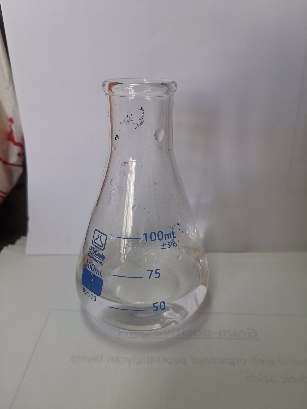 | 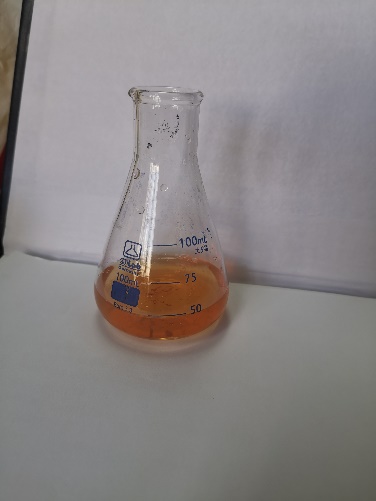 | 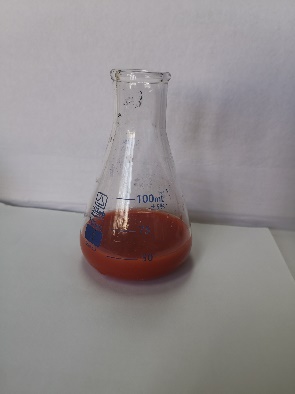 |
| --- | --- | --- |
| Fig. S5. green synthesis production of EPS-SeNPs the color change from colorless to dark orange | | |

| **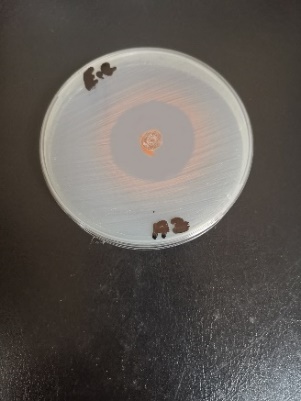** | **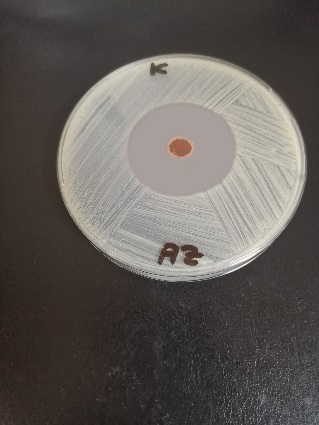** | **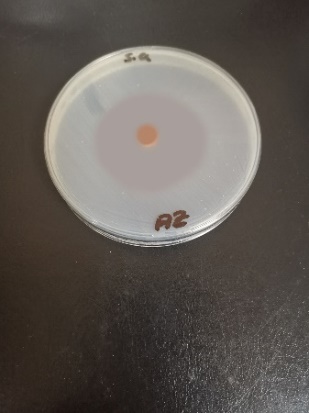** | **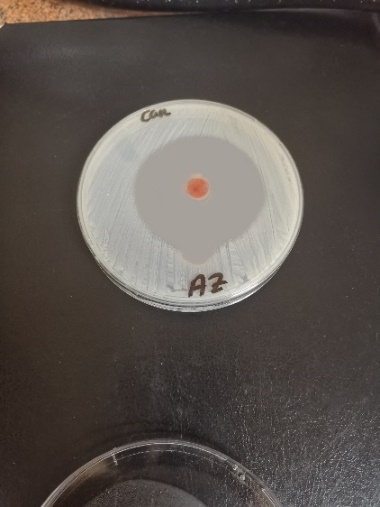** |
| --- | --- | --- | --- |
| ***E.coli*** | ***Klebsiella pneumoniae*** | ***Staphylococcus aureus*** | ***Candida albicans*** |
| Fig. S6: showing antimicrobial activity of AZEPS-SeNPs against *E. coli, Klebsiella pneumoniae*, *Staphylococcus aureus* and Candida *albicans* | | | |

**Table S1. Levels of independent variables in a Taguchi array**

| **Factors** | **Independent variables** | **Levels** | | |
| --- | --- | --- | --- | --- |
|  |  | **1** | **2** | **3** |
| **X1** | **Glucose (g/L)** | 10.0 | 20.0 | 30.0 |
| **X2** | **Mannitol (g/L)** | 10.0 | 20.0 | 30.0 |
| **X3** | **CaCl_2_ (g/L)** | 0.01 | 0.015 | 0.02 |
| **X4** | **MgSO_4_ (g/L)** | 0.01 | 0.015 | 0.020 |
| **X5** | **NaMoO_4_ (mg/L)** | 5.0 | 6.0 | 7.0 |
| **X6** | **K_2_HPO_4_ (g/L)** | 0.5 | 1.0 | 1.5 |
| **X7** | **KH_2_PO_4_ (g/L)** | 0.5 | 0.9 | 1.13 |
| **X8** | **FeSO_4_ (g/L)** | 0.010 | 0.012 | 0.014 |
| **X9** | **CaCo_3_ (g/L)** | 3.0 | 5.0 | 7.0 |
| **X10** | **pH** | 7.0 | 7.5 | 8.0 |
| **X11** | **Temp (ºC)** | 25.0 | 30.0 | 35.0 |
| **X12** | **Inoculum size (mL)** | 1.0 | 2.0 | 3.0 |
| **X13** | **Culture volume (mL)** | 25.0 | 50.0 | 75.0 |

**Table S2: Morphological, physiological, and biochemical characteristics isolated Azotobacter isolates.**

| No. | Source | Isolate Code | Colony morphology | Gram Stain | Cell shape | Biochemical parameter | | Colony color on Congo red agar |
| --- | --- | --- | --- | --- | --- | --- | --- | --- |
|  |  |  |  |  |  | **Catalase activity** | **Coagulase activity** |  |
| 1 | **Bean**  **(Vicia faba)** | AZ.1 | Entire, circular white to creamy color | Gram negative | Short bacilli | positive | positive | black |
| 2 |  | AZ.2 | Entire, circular white to creamy color | Gram positive | cyst | positive | positive | black |
| 3 |  | AZ.3 | Entire, circular white to creamy color | Gram negative | cyst | positive | positive | black |
| 4 |  | AZ.4 | Entire, circular white to creamy color | Gram negative | short bacilli | positive | positive | dark red |
| 5 |  | AZ.5 | Entire, circular white to creamy color | Gram negative | Coccobacilli | positive | positive | black |
| 6 |  | AZ.6 | Entire, circular white to creamy color | Gram positive | Short bacilli | positive | positive | black |
| 7 | **Wheat**  **)Triticum)** | AZ.7 | Entire, circular white to creamy color | Gram negative | Coccobacilli | positive | positive | black |
| 8 | **sweet potato** | AZ.8 | Entire, circular white to creamy color | Gram negative | Short bacilli | positive | positive | dark red |
| 9 | **Clover (Trifolium)** | AZ.9 | Entire, circular white to creamy color | Gram positive | Coccobacilli | positive | positive | dark red |
| 10 | **Garlic** | AZ.10 | Entire, circular white to creamy color | Gram negative | Coccobacilli | positive | positive | black |
| AZ :(Azotobacter species) | | | | | | | | |

**Table S3. Antimicrobial activity against tested pathogens.**

| **Tested Strains**  Isolates | **Inhibition zone diameter (mm)** | | | | | | |
| --- | --- | --- | --- | --- | --- | --- | --- |
|  | ***E. coli*** | ***Ent. aerogenes*** | ***S. aureus*** | ***K. pneumoniae*** | ***P. vulgaris*** | **MRSA** | ***Candida albicans*** |
| **AZ.1** | R | R | R | R | R | 6.0 | 7.0 |
| **AZ.2** | 7.0 | R | R | R | R | R | R |
| **AZ.3** | 6.0 | R | 5.5 | R | R | 7.0 | R |
| **AZ.4** | R | R | R | R | 7.0 | 7.0 | 6.0 |
| **AZ.5** | 5.5 | R | 5.5 | 7.0 | R | 6.0 | R |
| **AZ.6** | 8.0 | 7.0 | 6.0 | 7.0 | R | 5.5 | 8.0 |
| **AZ.7** | 6.0 | R | R | 6.0 | R | 6.0 | R |
| **AZ.8** | 7.0 | 6.0 | R | 5.5 | R | 6.0 | 6.0 |
| **AZ.9** | R | 6.0 | R | 6.0 | R | 5.5 | R |
| **AZ.10** | R | R | R | R | R | R | R |
| R: resistant | | | | | | | |

**Table S4. Response Table for Signal to Noise Ratios and means for EPS production.**

| **Table S4 (a). Response Table for Signal to Noise Ratios for variables Larger is better** | | | | | | | | | | | | | |
| --- | --- | --- | --- | --- | --- | --- | --- | --- | --- | --- | --- | --- | --- |
| **Level** | **Glucose** | **mannitol** | **CaCl2** | **MgSO4** | **NaMOo4** | **K2HPO4** | **KH2PO4** | **FeSO4** | **CaCo3** | **pH** | **Temp** | **Inoculum size** | **Culture volume** |
| **1** | 7.109 | 7.272 | 8.769 | 8.533 | 9.411 | 10.645 | 10.300 | 11.835 | 12.030 | 10.158 | 12.410 | 11.443 | 10.400 |
| **2** | 10.673 | 10.522 | 10.635 | 10.944 | 10.331 | 10.604 | 10.893 | 9.427 | 10.516 | 10.584 | 10.562 | 11.015 | 10.735 |
| **3** | 13.075 | 13.063 | 11.453 | 11.379 | 11.115 | 9.609 | 9.664 | 9.595 | 8.310 | 10.115 | 7.885 | 8.399 | 9.721 |
| **Delta** | 5.966 | 5.790 | 2.685 | 2.846 | 1.705 | 1.036 | 1.229 | 2.408 | 3.720 | 0.469 | 4.524 | 3.044 | 1.014 |
| **Rank** | 1 | 2 | 7 | 6 | 9 | 11 | 10 | 8 | 4 | 13 | 3 | 5 | 12 |
| **Table S4 (b). Response Table for variables Means** | | | | | | | | | | | | | |
| **Level** | **Glucose** | **mannitol** | **CaCl2** | **MgSO4** | **NaMoO4** | **K2HPO4** | **KH2PO4** | **FeSO4** | **CaCo3** | **pH** | **Temp** | **Inoculum size** | **Culture volume** |
| **1** | 2.799 | 2.793 | 3.371 | 3.429 | 3.166 | 3.633 | 3.724 | 4.000 | 4.166 | 3.468 | 4377 | 3.970 | 3.497 |
| **2** | 3.633 | 3.637 | 3.794 | 3.716 | 3.560 | 3.751 | 3.686 | 3.533 | 3.702 | 3.751 | 3689 | 3.736 | 3.782 |
| **3** | 4.613 | 4.616 | 3.880 | 3.901 | 4.320 | 3.661 | 3.636 | 3.512 | 3.178 | 3.827 | 2980 | 3.340 | 3.767 |
| **Delta** | 1.814 | 1.822 | 0.509 | 0.472 | 1.154 | 0.118 | 0.089 | 0.488 | 0.988 | 0.359 | 1397 | 0.630 | 0.286 |
| **Rank** | 2 | 1 | 7 | 9 | 4 | 12 | 13 | 8 | 5 | 10 | 3 | 6 | 11 |

**Table S5.** **Analysis of Variance for EPS production**

|  | **EPS production** | | | | | |
| --- | --- | --- | --- | --- | --- | --- |
| **Source** | **DF** | **Seq SS** | **Adj MS** | **F** | **P** | **% Contribution** |
| Regression | 13 | 54.8938 | 4.2226 | 51.95 | 0.000 |  |
| Glucose | 1 | 14.8149 | 14.8149 | 182.28 | 0.000 | 26.478 |
| mannitol | 1 | 14.9422 | 14.9422 | 183.85 | 0.000 | 26.706 |
| CaCl_2_ | 1 | 1.1654 | 1.1654 | 14.34 | 0.002 | 2.0829 |
| MgSO_4_ | 1 | 1.0035 | 1.0035 | 12.35 | 0.004 | 1.7935 |
| NaMOo_4_ | 1 | 5.9973 | 5.9973 | 73.79 | 0.000 | 10.129 |
| K_2_HPO_4_ | 1 | 0.0035 | 0.0035 | 0.04 | 0.839 | 0.0062 |
| KH_2_PO_4_ | 1 | 0.0339 | 0.0339 | 0.42 | 0.529 | 0.0605 |
| FeSO_4_ | 1 | 1.0707 | 1.0707 | 13.17 | 0.003 | 1.9136 |
| CaCo_3_ | 1 | 4.3907 | 4.3907 | 54.02 | 0.000 | 7.8474 |
| pH | 1 | 0.5796 | 0.5796 | 7.13 | 0.019 | 1.0359 |
| Temp | 1 | 8.7780 | 8.7780 | 108.00 | 0.000 | 15.688 |
| Inoculum size | 1 | 1.7860 | 1.7860 | 21.98 | 0.000 | 3.192 |
| Culture volume | 1 | 0.3280 | 0.3280 | 4.04 | 0.066 | 0.005 |
| Error | 13 | 1.0566 | 0.0813 |  |  | 1.888 |
| Total | 26 | 55.9504 |  |  |  | 100 |
| DF, The total degrees of freedom. Seq SS, Sequential sums of squares. Adj MS, Adjusted sums of squares. F, F-value. P, p-value. | | | | | | |

**Table S6. Analysis of Variance regression for EPS production Coefficients (EPS production)**

| **Term** | **Coef** | **SE Coef** | **T-Value** | **P-Value** | **VIF** |
| --- | --- | --- | --- | --- | --- |
| Constant | 844 | 487 | 1.73 | 0.107 |  |
| Glucose | 907.2 | 67.1 | 13.51 | 0.000 | 1.00 |
| mannitol | 911.1 | 67.1 | 13.57 | 0.000 | 1.00 |
| CaCl_2_ | 254.4 | 67.1 | 3.79 | 0.002 | 1.00 |
| MgSO_4_ | 236.1 | 67.1 | 3.52 | 0.004 | 1.00 |
| NaMoO_4_ | 577.2 | 67.1 | 8.60 | 0.000 | 1.00 |
| K_2_HPO_4_ | 13.9 | 67.1 | 0.21 | 0.839 | 1.00 |
| KH_2_PO_4_ | -44.4 | 67.1 | -0.66 | 0.520 | 1.00 |
| FeSO_4_ | -243.9 | 67.1 | -3.63 | 0.003 | 1.00 |
| CaCo_3_ | -493.9 | 67.1 | -7.36 | 0.000 | 1.00 |
| pH | 179.4 | 67.1 | 2.67 | 0.019 | 1.00 |
| Temp | -698.3 | 67.1 | -10.40 | 0.000 | 1.00 |
| Inoculum size | -315.0 | 67.1 | -4.69 | 0.000 | 1.00 |
| Culture volume | 135.0 | 67.1 | 2.01 | 0.066 | 1.00 |

**Table S7: The therapeutic index and IC50 of AZEPS, AZEPS-SeNPs and Staurosporine against A549, WI38**

| **Sample** | **Cytotoxicity, IC50 (µg/mL)** | | **Therapeutic index** |
| --- | --- | --- | --- |
|  | **A549** | **WI38** | **WI38/ A549** |
| **AZEPS** | 17.49±0.85 c | 41.56±1.83 c | 2.37±0.11^a^ |
| **AZEPS-SeNPs** | 1.724±0.08 a | 12.39±0.54 a | 7.18±0.21^c^ |
| **Staurosporine** | 6.435±0.31 b | 27.17±1.19 b | 4.22±0.23^b^ |

Different letters a, b and c within the same column indicate that they are significantly different at p<0.05 (letter a is the smallest, followed by b and finally the letter c is the highest one).

**Table S8: A549 cells death as affected by AZEPS and AZEPS-SeNPs.**

|  | **Sample** | **Apoptosis %** | | | **Necrosis%** |
| --- | --- | --- | --- | --- | --- |
|  |  | **Total** | **Early** | **Late** |  |
| **1** | **AZEPS /A549** | 33.72 | 8.98 | 17.59 | 7.15 |
| **2** | **AZEPS-SeNPs /A549** | 53.76 | 15.43 | 35.28 | 3.05 |
| **3** | **Cont.A549** | 2.43 | 0.51 | 0.13 | 1.79 |

**Table S9: AZEPS and AZEPS-SeNPs influence cell cycle progression in A549 cells.**

| **Sample** | **DNA content** | | |  |
| --- | --- | --- | --- | --- |
|  | **%G0-G1** | **%S** | **%G2/M** | **Comment** |
| **AZEPS /A549** | 54.61 | 38.76 | 6.63 | cell growth arrest@ S |
| **AZEPS-SeNPs /A549** | 46.36 | 52.04 | 1.6 | cell growth arrest@ S |
| **Cont.A549** | 59.04 | 33.73 | 7.23 |  |
